# Identifying Effective Biosecurity Measures for Preventing the Introduction of Classical Swine Fever in Pig Farms in Japan: Under the Condition of Absence/Presence of Observable Infected Wild Boar

**DOI:** 10.1155/2024/1305664

**Published:** 2024-07-31

**Authors:** Makoto Ukita, Ryota Matsuyama, Norikazu Isoda, Ryosuke Omori, Takehisa Yamamoto, Kohei Makita

**Affiliations:** ^1^Veterinary Epidemiology Unit, Graduate School of Veterinary Medicine, Rakuno Gakuen University, 582 Bunkyodai Midorimachi, Ebetsu 069-8501, Hokkaido, Japan; ^2^Laboratory of Microbiology, Department of Disease Control, Faculty of Veterinary Medicine, Hokkaido University, Kita 18, Nishi 9, Kita-Ku, Sapporo 060-0818, Hokkaido, Japan; ^3^Division of Bioinformatics, International Institute for Zoonosis Control, Hokkaido University, Kita 20 Nishi 10, Kita-Ku, Sapporo 001-0020, Hokkaido, Japan; ^4^Epidemiology and Arbovirus Group, Division of Transboundary Animal Disease Research, National Institute of Animal Health, National Agriculture and Food Research Organization, 3-1-5 Kannondai, Tsukuba 305-0856, Ibaraki, Japan

## Abstract

The outbreak of infectious diseases in swine, such as classical swine fever (CSF), has become a significant concern in the pig-farming industry. In Japan, after the re-emergence of CSF in 2018, farms are now exposed to the risk of transmission from infected wild boar and CSF-contaminated farms. This study aimed to identify biosecurity measures that were effective for the prevention of CSF introduction into farms during the period from the beginning of the CSF epidemic to the implementation of a vaccination campaign for domestic pigs at risk. The probability of virus introduction was assumed to be increased by the elevated risk from CSF-infected wild boar and infected farms around the farm. The risk from infected wild boar was represented by the prevalence of CSF in wild boar or the occupancy of 1-km grid cells with infected wild boar within 10-km radii from a pig farm and the occurrence of CSF outbreaks on neighboring farms. Conversely, the probability of virus introduction was assumed to decrease in response to on-farm biosecurity measures being implemented on each farm. The implementation of biosecurity measures on the farms and farm attributes were obtained through a questionnaire survey. Analyses were performed on each farm under the weekly situations where infected wild boar were both absent and present in the vicinity using a binomial generalized linear model. On farms where infected wild boar were not present around farms, daily washing and disinfecting of work clothing in pig houses was identified as the main measure to reduce the risk of CSF introduction into farms. On farms with infected wild boar in the vicinity, the absence of public roads on the farm and preventing wildlife intrusion into the areas where pig carcasses were stored were demonstrated to be effective in preventing CSF introduction. Based on the assumption that strict and comprehensive biosecurity measures are required to prevent CSF introduction, the implementation of these potentially effective measures is worth being prioritized.

## 1. Introduction

Classical swine fever (CSF), caused by the CSF virus (CSFV), a single-stranded RNA virus of the *Pestivirus* genus of the *Flaviviridae* family, is an important infectious disease in pigs due to its high transmissibility and mortality. The disease is designated as a notifiable swine disease by the World Organisation for Animal Health (WOAH) [1]. Epidemics of the disease cause significant economic losses in affected countries due to losses in livestock production and the costs incurred for prevention, control, and eradication [2], as well as restriction of the trade of animals and their products [3]. For example, a CSF epidemic in the Netherlands between 1997 and 1998 affected a total of 429 farms and resulted in the loss of US$ 2.3 billion [4]. In the case of Colombia, the economic losses attributed to a CSF epidemic in 2015–2016 were reported to be US$ 3.8 million [2]. Vaccination and the implementation of various biosecurity measures have been conducted in the affected countries to prevent the transmission of disease [3]. Vaccination prevents disease occurrence and spread on farms. Biosecurity measures on farms, in addition to these, are important to prevent the disease introduction from outside and within-farm spread.

Inappropriate biosecurity practices have often been identified as the cause of pathogen introduction into farms [5, 6]. Regarding CSF, a distinction is made between direct (from pig to pig) and indirect (using one or more intermediate steps) spread. Indirect transmission routes are further split between distance-independent and distance-dependent transmission routes into the farm [7]. Distance-independent transmission includes direct and indirect contact with wild boar-derived viruses and mechanical transmission, such as via artificial insemination or swill feeding [7]. During the CSF epidemics in Europe in the 1990s, distance dependency in the risk of transmission between farms was investigated extensively [8, 9]. Vehicles, birds, rodents, and airborne transmission have also been cited as possible sources of neighbor-to-neighbor transmission between farms [10, 11]. The attribute of farms is another risk factor that is involved with the risk of CSF introduction into farms. For example, as an environmental attribute, the presence of public roads on the farm enabling the crossing of farm staff and equipment were also noted as risk factors for the occurrence of CSF outbreak [12, 13]. Also, adjacency to a forest as an environmental farm attribute is considered to increase the risk of the transmission of infectious diseases from wildlife [14].

In particular, the transmission of devastating swine infectious diseases from wild boar to domestic pigs has become a significant concern in the pig-farming industry. The wild boar population acts as a reservoir for CSF, and wild boars are frequently responsible for the transmission of the disease to domestic pigs through direct or indirect contact [7, 15]. A previous study reported that approximately 60% of CSF outbreaks on pig farms in Germany in the 1990s were caused by direct or indirect contact with wild boar [16]. Similarly, in the current CSF epidemic in Japan, the introduction of CSFV from wild boar through direct or indirect contact is considered to be the primary cause of outbreaks on farms [12, 13]. Wild boar populations also play a pivotal role in the transmission of African swine fever (ASF) [6]. In Europe, where ASF is endemic in wild boar, it was suggested that perimeter fences should be erected on farms to mitigate both direct and indirect contact with wild boar [17].

In the case of the CSF epidemic in Japan, the absence of biosecurity measures, such as inadequate disinfection of vehicles or the failure to change boots and clothing when entering pig houses, was widely suggested as a possible cause of CSFV introduction into farms [12, 13]. The implementing effective biosecurity measures is thus considered to be key to controlling the CSF epidemic. In Japan, farms are required to implement biosecurity measures based on the Biosecurity Standards defined by the National Act on the Prevention of Infectious Diseases in Livestock (Act No. 166 of May 31, 1951) [18]. The law provides for penalties if a farmer violates the standards and does not comply with improvements. Given this legal requirement, compliance with the standards has been comparatively high; for instance, compliance with the 34 biosecurity measures prescribed by the standards in 2018 reached 80%–90% in that year [19]. In addition, some farmers implemented biosecurity measures voluntarily, such as erecting electric fencing around their farms to prevent the transmission of CSFV from wild boar. In areas where CSF-infected wild boar were detected, the provision of perimeter fence and the use of special clothing for each pig house were also encouraged by the veterinary authority [20]. The occurrence of CSF outbreaks despite these farmers' efforts implies that the biosecurity measures might not be sufficiently effective in preventing CSF transmission. The effectiveness of the biosecurity measures should, therefore, be evaluated, considering the process of introduction of CSF into farms. Incidentally, after this CSF outbreak, the Biosecurity Standards were revised to strengthen precautions, such as adequate prevention measures for wild animals.

This study aimed to identify which biosecurity practices or farm attributes are effective for preventing outbreaks of CSF on farms in Japan. To achieve this aim, statistical models were developed assuming (i) the transmission of CSFV to pig farms from infected wild boar and other infected farms and (ii) the prevention of CSF on pig farms by implementing biosecurity measures and by the farm attributes that decrease the risk of transmission. The association of farm attributes and the effectiveness of biosecurity measures against CSF outbreaks were then examined relative to the presence or absence of infected wild boar around farms.

## 2. Materials and Methods

In the present study, the weekly probability of CSFV introduction to a susceptible farm was modeled by assuming that (i) the existence of infected wild boar in the vicinity of the farm and CSF outbreaks on neighboring farms both increase the probability of virus introduction and (ii) the implementation of biosecurity measures and the farm attributes that positively affects the prevention of disease on a farm decreases the probability of virus introduction. Using the model, the risk of CSFV introduction from wild boar and neighboring farms, and the preventive effect of biosecurity measures were estimated. To achieve this, data on the farm attributes and the implementation status of farm biosecurity measures were collected by means of a postal questionnaire survey. In addition, epidemiological data on CSF among captured wild boar were extracted from the national CSF surveillance data for wild boar. Furthermore, epidemiological data on the CSF outbreaks on neighboring farms were extracted from the reports of epidemiological investigations conducted by the Ministry of Agriculture, Forestry, and Fisheries (MAFF) [12, 13]. The risk of CSFV transmission from wild boar and from neighboring farms, as well as the preventive effect of specific biosecurity measures, were estimated by binomial regression. The details of these calculations are described in the following sections.

### 2.1. Study Period

This study focused on the preventive effect of biosecurity measures for CSF from the beginning of the CSF epidemic to the implementation of a vaccination campaign for domestic pigs at risk. In the current CSF epidemic in Japan, the first CSF case of a domestic pig was reported in Gifu Prefecture on September 9, 2018. Subsequently, through active surveillance conducted by the Gifu Prefectural Government, CSF infection in wild boar was reported on September 13, 2018 [14, 21]. As the area affected by the epidemic has expanded, preventive vaccination for domestic pigs has been implemented in areas adjacent to the epidemic area since October 25, 2019 [14, 21, 22]. Thus, the research period spanned from September 9, 2018 to October 26, 2019, a total of 59 weeks: the weekly interval periods starting from the initiation of the wild boar surveillance program to the commencement of the pig vaccination campaign on pig farms. The period after October 26, 2019 was not included because of the potentially strong effect of the vaccination campaign on the farm susceptibility against CSFV, which may mask the preventive effects of the biosecurity practice against the virus.

### 2.2. Data Collection and Processing

#### 2.2.1. Postal Questionnaire Survey for Assessing Farm Attributes and the Implementation of Biosecurity Measures

A questionnaire based on the Biosecurity Standards and a previous study [23] was designed to collect information on the farm attributes and the biosecurity practices implemented on pig farms during the CSF outbreaks. The first version of the questionnaire was drafted with the cooperation of four veterinarians with more than 5 years of experience in swine clinical veterinary services in the epidemic area. A pilot survey was then conducted with seven pig farmers who were not included in the present study, and the questionnaire was subsequently modified according to their comments. The final version of the questionnaire consisted of questions on eight categories: (i) farmer's attributes; (ii) farm attributes; (iii) biosecurity practices employed when farm staff enter and exit the farm area, hygiene control area (i.e., an area on the farm that is established to prevent the entry and/or spread of pathogens and which is separated from other areas by a clearly defined border that is demarcated using fences), and pig houses; (iv) biosecurity practices for rearing pigs, (v) biosecurity practices for feed and drinking water for pigs, (vi) biosecurity practices regarding farm staff, (vii) biosecurity practices when the farm was visited by persons other than farm employees (feed transport services, veterinarians, facility construction services, and other outside visitors), and (viii) biosecurity practices to prevent intrusion of wildlife (Table 1). All of the questions focused on the status of the biosecurity practices that were employed when the initial CSF case was reported in the prefecture where the respondent's farm was located. Most of the questions about biosecurity practices employed a five-point Likert scale (1: never/very poor, 2: rarely/poor, 3: sometimes/neither good nor poor, 4: often/good, and 5: always/very good), and some questions required respondents to supply a numerical value or a “yes/no” response (Table 1).

**Figure 1:**
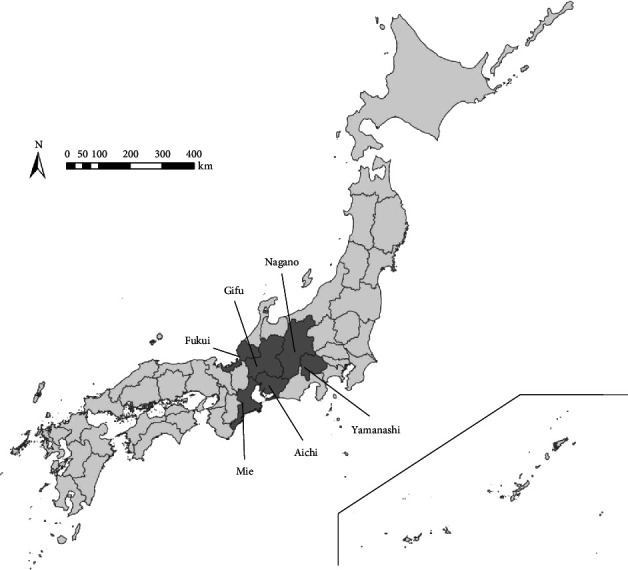
Prefectures with CSF outbreaks on pig farms that occurred between September 2018 and October 2019 (dark gray area). Questionnaire surveys were conducted in these six prefectures.

The postal survey was conducted in six prefectures in Japan (Aichi, Fukui, Gifu, Mie, Nagano, and Yamanashi Prefectures; Figure 1) under the cooperation of prefectural governments and ensuring the anonymity of the respondents. The survey was approved by the Research Ethical Committee of Rakuno Gakuen University (Approval No: 20-5). An ethics statement was provided on the questionnaire, and informed consent was implied by participation in the survey. The questionnaires were distributed from May to June 2021 to all farms where CSF introduction had occurred between September 2018 and October 2019 (i.e., infected farms) and all farms located within a 10-km radius of the infected farms (i.e., noninfected farms), except farms that had been closed or farms that kept feed pigs as pets. Farms within a 10-km radius of an infected farm were assumed to have a similar risk of exposure to infected wild boars, with reference to the habitat ecology of wild boars [24]. Saitama and Gunma Prefectures, which had CSF outbreaks on farms during this period, were excluded because the CSF infection among wild boar in these prefectures was detected only around October 2019, which was the end of the study period. Responses to the questionnaires were returned to Rakuno Gakuen University by the end of October 2021. The reason for using this postal survey is to avoid inaccurate responses due to respondents' concern about penalties based on the content of their responses, as is the case when public veterinarians visit farms for face-to-face interviews. All responses were then recorded in a database (Microsoft® Access, Microsoft Inc., Redmond, WA). The response rate of the questionnaires is shown in Table 2.

#### 2.2.2. Data Processing of Responses to the Questionnaire

For further analyses using the binomial regression model (Section 2.3), (i) farms that responded to less than 70% of the survey questions and (ii) farms that did not provide sufficient information to identify their location were excluded from the analysis. As a result, 53 farms in four prefectures (Aichi, Gifu, Mie, and Nagano) were included in the analysis.

Answers to items in the questionnaire with a five-point Likert scale for the practice of biosecurity measures were divided into binary variables: 1–3, suggesting not conducted or not sure, was converted into 0, and 4 and 5, suggesting conducted, into 1. The height of the perimeter fence surrounding the farm was categorized into two categories of ≥1.5 and <1.5 m based on the jumping ability of wild boar [25]. The mesh size of the perimeter fence was classified into two categories of 5 or <5 cm based on the size to prevent intrusion of wild boar piglets and medium-sized wild animals [26, 27]. The mesh size of the bird net was categorized into two categories of <2 and ≥2 cm, based on the size requirements specified in the Biosecurity Standards of Japan [18]. In addition, items with extremely high implementation rates were excluded from the analyses because their effect could not be estimated. Some omissions in the responses were found; the average missing rate was 4.1% (range: 0.0%−13.2%). These missing values were imputed using a random forest imputation algorithm implemented in the missForest package [28] in R.

#### 2.2.3. Epidemiological Characteristics of Pig Farms

The farms were classified into three types based on the CSF outbreak status: infected, noninfected, and epidemiologically related. Infected and noninfected farms were defined as those on which CSF outbreaks did or did not occur during the study period. The epidemiologically related farm was defined as a farm that did not have an outbreak and culled pigs on the farm according to at least one of the following conditions: (i) located in a pig-farm-complex which included an infected farm, (ii) had introduced pigs from an infected farm, and (iii) geographically separated from but affiliated with an infected farm in terms of farm management.

The date of introduction of CSFV in the infected farms and the duration of viral shedding from the farms were defined based on the reports of CSF epidemiological investigations conducted by MAFF [12, 13]. In the reports, 41 outbreaks were reported in the study area during the study period. The date when the virus was introduced into each infected farm was estimated and described by MAFF based on the results of enzyme-linked immunosorbent assay and polymerase chain reaction (PCR) analyses at the time of culling [12, 13, 29]. The median date of the estimated range was used as the date of introduction on each farm. Then, the duration of viral shedding from the outbreak farm was assumed to be between the estimated date of CSFV introduction and the date when the culling of all pigs on the farm was completed.

Furthermore, forest coverage around each farm was included as an additional explanatory variable to describe farm attributes. It was assumed that forests act as interfaces between wild boar and pigs and that their coverage represents areas of possible contact. Farms were categorized into “forest area” if more than 50% of the area within a 500-m buffer around each farm was covered by forest, which was estimated using 100-m mesh land-use data, i.e., National Spatial Data provided by the Ministry of Land, Infrastructure, Transport and Tourism [30]. Farms that did not satisfy this criterion were classified as a “non-forest area.” This binary categorization was performed using ArcGIS Pro 3.0.0 software (ESRI Inc., Redlands, CA).

#### 2.2.4. Surveillance Data for Wild Boar

**Figure 2:**
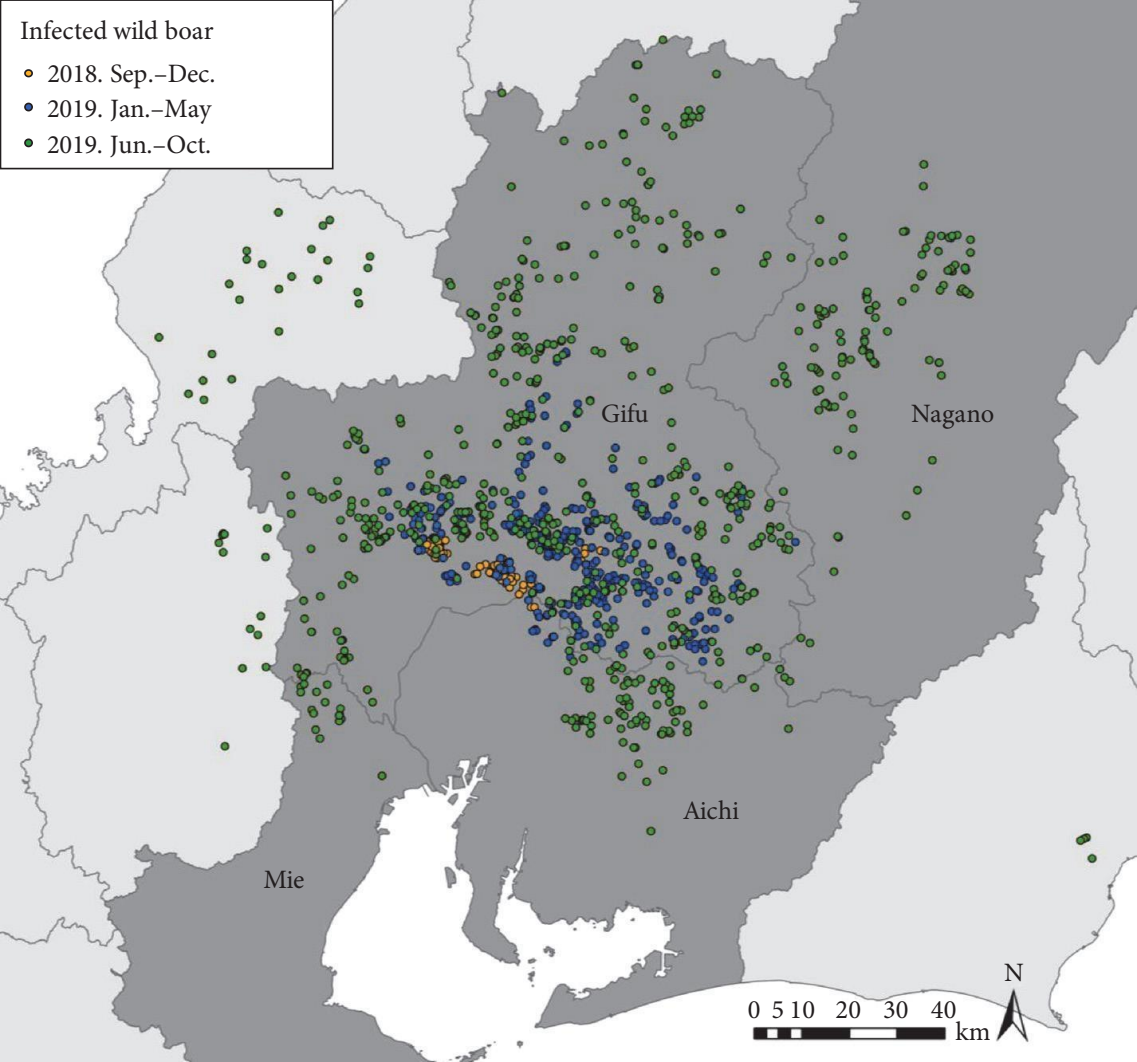
Location of infected wild boar detected during the analysis period in Aichi, Gifu, Mie, and Nagano prefectures. The orange, blue, and green symbols denote infection during September–December in 2018, January–May in 2019, and June–October in 2019, respectively.

To date, the surveillance of CSF in wild boar populations has been conducted by prefectural governments, and the surveillance results have been reported to MAFF. The complete surveillance data for the targeted prefectures were obtained from MAFF (i.e., Aichi, Gifu, Nagano, and Mie Prefectures) in the targeted study period (i.e., between September 9, 2018 and October 26, 2019). The data include PCR test results for CSF infection, locations (longitude and latitude), and detection dates of the wild boar captured. From September 2018 to October 2019, the number of wild boar tested in Aichi, Gifu, Mie, and Nagano Prefectures was 1,303, 2,533, 728, and 439, respectively, and of these, 101, 1,061, 21, and 126 were infected, respectively. The distribution and the timing of detection of infected wild boar are shown in Figure 2.

### 2.3. Modeling the Risk of Transmission from Infected Farms and Wild Boar to Pig Farms

The data for the outbreak farms and the infected wild boar were arranged into weekly time-series data. The probability of infection for farm *i* in week *t* was modeled by considering (i) transmission risk from infected wild boar and infected farms and (ii) the preventive effect of farm attributes and biosecurity measures that farm *i* employed. The details of these components are described below.

The transmission risk from infected farms was assumed to be affected by the distance between the susceptible and infected farms. The distance between farms was classified into two categories, greater or less than 3 km, by referring to the radius of the “movement restriction zone” designated as the area where the movement of domestic animals is prohibited by the Act on Domestic Animal Infectious Diseases Control (Act No. 166 of May 31, 1951). This categorization was employed because the movement restriction zone was determined as the high-risk area of transmission from the infected farm. Referring to *f*_*i*,*t*_ as the number of infected farms within a 3-km radius of farm *i* at week *t*, the transmission risk on the farm *i* at week *t*, *frisk*_*i*,*t*_, is assumed as follows:

(1) $$\begin{array}{c} {frisk}_{i,t}\propto f_{i,t}. \end{array}$$

Concerning modeling the transmission risk from wild boar to farms, fine-scale data on the population density of wild boar and those on surveillance efforts were not available for this study area. As a result, accurate density and distribution estimates of infected wild boar around farms are unavailable. Two modeling approaches were therefore employed to resolve this limitation. In the first approach, the transmission risk from wild boar was modeled to be proportional to the prevalence of infected wild boar around a farm. This approach assumes that the transmission risk from wild boar is not entirely dependent upon the number of infected animals per unit area but on the proportion of infected animals in the area. This approach has an advantage in the stability of prevalence under the already known circumstance that the surveillance efforts are not equal among locations and time series due to the accessibility in terms of ecological settings (deep forest or snow), availability of skilled hunters, and difference of the prefectures. In such circumstances, the proportion of infected animals serves as a relatively robust indicator for modeling the risk of disease transmission as compared to the number of infected animals [31, 32].

In the second approach, the risk of transmission from wild boar was modeled by the distance from the geographical area where infected animals were found. This assumes that the habitat of infected wild boars identified in the surveillance program is correlated with the number of infected wild boars and that the distance from the habitat affects the risk of transmission. This approach was adopted because the surveillance data accurately reflected the number and distribution of infected wild boar; the transmission risk could, therefore, be explicitly modeled using the surveillance data and a distance-based transmission kernel. In addition, by using this approach, it is assumed that areas where infected wild boar were detected would continue to pose a transmission risk until the conclusion of the study period. This assumption is rational if infected wild boar are detected continuously within the same area and if CSFV remains viable for an extended period in carcasses of infected wild boar and/or other possible vectors in the environment [7, 33, 34]. This approach has also been adopted elsewhere [35, 36].

The transmission risk from wild boar was modeled based on the infectious status of wild boar within a 10-km radius of each farm with reference to the habitat ecology of wild boar [24]. The first modeling approach calculated the weekly prevalence of CSF among wild boar around each farm (*Supplementary figure 1*). Since the small sample size in some weeks resulted in a prevalence in an area that is strongly influenced by the results of a single sample, a 3-week centered moving average was used for each area (*Supplementary figure 2*). Accordingly, in the first modeling approach (hereinafter, referred to as the prevalence approach), the transmission risk of farm *i* by infected wild boar at week *t* (*wrisk*_*i*,*t*_) can be defined as follows:

(2) $$\begin{array}{c} {wrisk}_{i,t}\propto prev_{i,t}, \end{array}$$

where *prev*_*i*,*t*_ denotes the 3-week moving average of the prevalence of CSF among wild boar around farm *i* in week *t*.

In the second modeling approach (hereinafter, referred to as the transmission kernel-based (TK) approach), the kernel around infected wild boar represented the distance-based risk. The spatial distribution of wild boar was described using a grid of 1 km cells, referring to the fact that a typical habitat area of Japanese wild boar is approximately 1 km^2^ [37]. Any 1-km grid cell where at least one infected wild boar was reported was defined as an infected grid cell (hereinafter, referred to as an infected cell). To account for the delay in detection based on previous studies, the infected cells were assumed to be infectious for 4 weeks before the week that the first infected wild boar was reported [32, 35, 38]. In addition, infected cells were assumed to retain transmissibility until the end of the study period, as assumed in a previous study [35]. Using the TK approach, which employs a transmission kernel previously estimated by Hayama et al. [35], the transmission risk of farm *i* from infected wild boar at week *t* (*wrisk*_*i*,*t*_) was modeled as a sum of hazards from infected cells up to week *t*, i.e.,

(3) $$\begin{array}{c} {wrisk}_{i,t}=\sum_{c\in infected\;cell}\left(1-\text{exp}\left(-{\left(\frac{r_{i,c}}{r_{0}}\right)}^{-\theta }\right)\right), \end{array}$$

where *r*_*i*,*c*_ represents the Euclidean distance between the centroid of an infected cell (*c*) and the farm *i*, *r*_0_ denotes the shape parameter of the transmission kernel, and *θ* denotes the scale parameter of the transmission kernel. In this study, the values of *r*_0_ and *θ* were fixed as described in Hayama et al. [35] (i.e., *r*_0_ = 1.29 and *θ* = 2.81).

The distribution of infected wild boars expanded gradually during the study period, causing changes in the risk of transmission and the effectiveness of control measures over time. It was assumed that the strength of biosecurity measures against the transmission risk from wild boar varied depending on whether infected wild boar were present or absent around the farm. Each farm status was classified as (i) wild boar-derived risk phase (WB phase) and (ii) non-wild boar-derived risk phase (non-WB phase), based on the infectious status of wild boar around the farm in week *t*. That is, before an infected wild boar was detected within a 10-km radius of the farm, the farm status was classified as being in the non-WB phase, but once an infected wild boar was detected, the farm status changed to the WB phase. The contribution of farm attributes and the effect of biosecurity measures on CSF transmission in the WB and non-WB phases were estimated separately.

Note that the reference time point for differentiating WB/non-WB status varied between the two modeling approaches. In the prevalence approach, the week when the 3-week moving average of the prevalence exceeded zero for the first time was taken as the reference time point. In other words, the time at 1 week before the infected wild boar was detected was considered the reference time point. Similarly, in the TK approach, the week when the *wrisk*_*i*,*t*_ exceeded zero at the first time was considered as the reference time point. In this case, the reference time point was 4 weeks before the actual time at which infected wild boar were detected.

### 2.4. Estimation of the Probability of Infection at Each Farm

The weekly probability of introduction of CSFV was estimated until an outbreak event occurred on the farm. The end of the analytical periods for noninfected farms was the end of the study period. In contrast, for epidemiologically related farms, it was the week in which the culling of pigs was completed. The probability of virus introduction on farm *i* at week *t* (*p*_*i*,*t*_) was modeled using the following equation:

(4) $$\begin{array}{c} p_{i,t}=1-\text{exp}\left\{-\text{exp}\left(\alpha_{0}+\alpha_{w}\;{wrisk}_{i,t}+\alpha_{f}\;{frisk}_{i,t}+\sum_{k}\;\beta_{k}{biosecurity}_{i,k}\right)\right\}, \end{array}$$

where *α*_0_, *α*_*w*_, *α*_*f*_, and *β*_*k*_ denote the intercept (i.e., the baseline hazard that is not influenced by infected wild boar, infected farms, and the implementation of biosecurity measures or farm attributes), the coefficient of the variable of infection risk from wild boar (*wrisk*_*i*,*t*_), the coefficient of the variable infection risk from infected farms (*frisk*_*i*,*t*_), and the coefficient of the preventive effect by the *k*th variable in biosecurity measure and farm attribute variables, respectively. Note that *p*_*i*,*t*_ is a probability with a value of between 0 and 1.

Estimation of the coefficients was conducted by binomial regression with a complementary-log-log (cloglog) link function, which is widely used to describe the hazard in the survival process in the framework of the generalized linear model (the glm function in *R*) [39]. The weekly escape/occurrences of CSF outbreaks on each farm were set as the response variable, and the coefficients of the variables in Equation (4) were estimated. The “escape of CSF outbreaks” denotes the successful prevention of CSF introduction into farms, whereas the “occurrence of CSF outbreaks” denotes the occurrence of CSF introduction. To evaluate the contribution of farm attributes and the efficacy of biosecurity measures using the limited data, first univariable analyses for 48 farm attributes and biosecurity measure variables were conducted. Then, multivariable analyses were performed using the selected variables from the univariable studies. The univariable analysis included the variables of infected wild boar-derived and/or farm-derived transmission risks in Equation (4) and employed only a single farm attribute or biosecurity variable. The term wild boar-derived risk was included only in the analyses on the WB phase, while that of infected farm-derived risk was included in either of the WB/non-WB phases. It was assumed that the farm attributes that positively influence the prevention and the effective biosecurity measures decreased the probability of infection in a farm. Thus, the variables with a negative coefficient and a *p*-value less than 0.2, estimated by the Wald test in the univariable analyses, were selected for subsequent multivariable analyses. The final model in the multivariable analyses was determined by selecting the model with the lowest akaike information criterion (AIC) calculated with the maximum likelihood estimates [40].

Multicollinearity between variables was confirmed by the variance inflation factor (VIF). A VIF value > 10 was considered to indicate a strong correlation between variables [41]. R version 4.2.0 was used for all statistical analyses [42].

## 3. Results

### 3.1. Status of CSF Outbreaks, Farm Attributes, and Implementation of Biosecurity Measures

**Figure 3:**
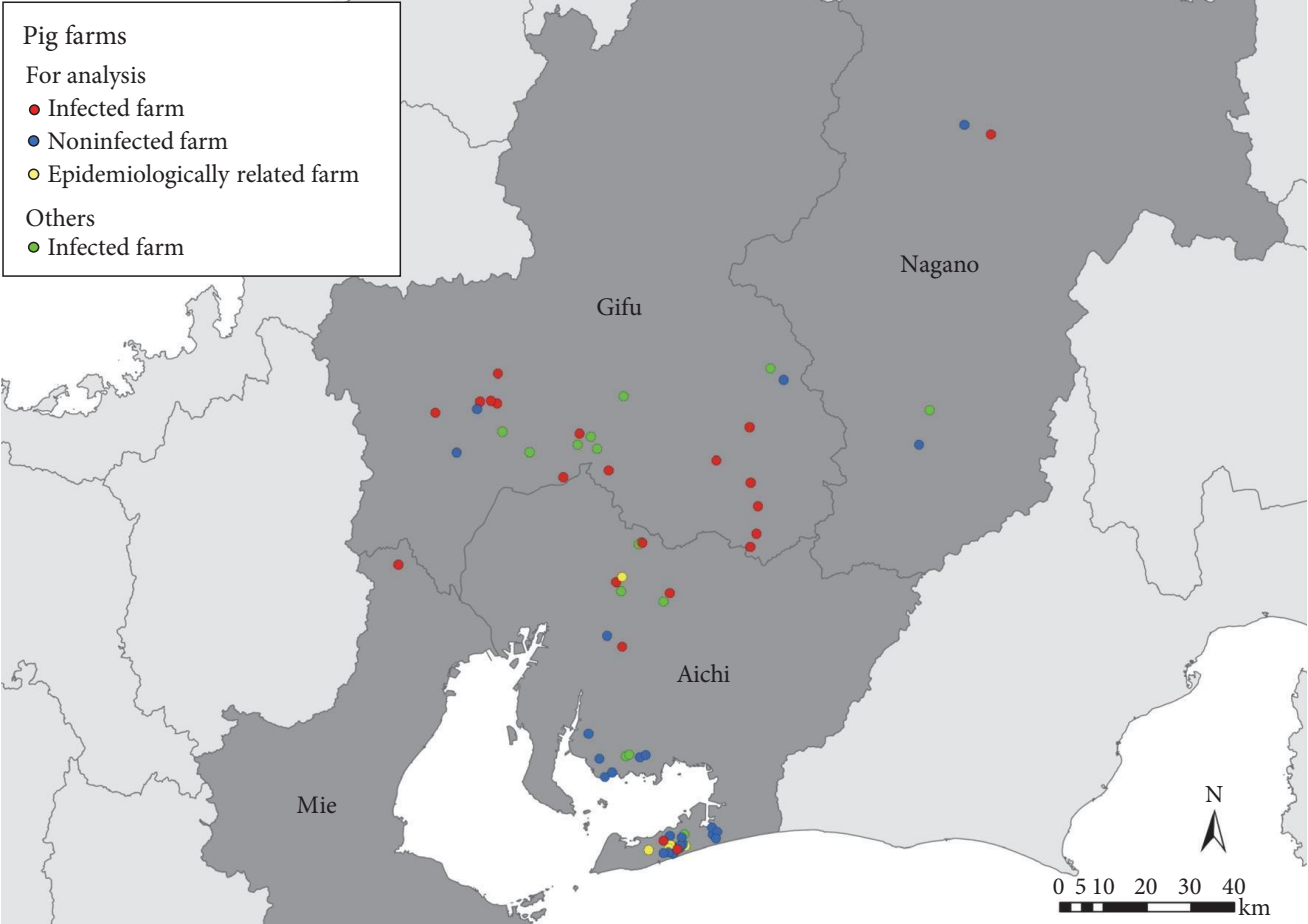
Pig farms considered in the analysis, and other infected farms identified between September 2018 and October 2019 in the four prefectures. The solid red, blue, and yellow circles denote infected, noninfected, and epidemiologically related farms (*n* = 21, 25, and 7) for the analysis. There were 41 outbreaks during this study period. The green circles denote the other infected farms (*n* = 20).

The locations of the 53 farms considered in the final analysis and the other infected farms are shown in Figure 3. Among the farms included in the final analysis, 21, 25, and 7 were classified as infected, noninfected, and epidemiologically related farms, respectively. Among the epidemiologically related farms, five were located in pig farm complexes with outbreak farms, two introduced pigs from outbreak farms, and one farm was affiliated with an outbreak farm.

A full list of the proportions of farms that meet the farm attributes and the implementation rates of biosecurity measures among the farms that responded to the questionnaires was provided in *Supplementary table 3*. The number of farrow-to-finish, fattening, and reproduction farms were 37, 11, and 3, respectively (*n* = 51). The proportion of farms located in a forest area was 39.6% (21/53). As for the biosecurity measures related to when farmers enter and leave the farm area, the implementation rate of “properly adjusting the concentration of disinfectant for vehicles” was 80.9% (38/47). However, the implementation rate of “parking staff's vehicles off-site or disinfecting vehicles before entering the farm site” was only 58.5% (31/53). The implementation rate of the biosecurity measures prescribed by the Biosecurity Standards in 2018, such as “restricting the entry of visitors not related to the farm” (90.6% (48/53)) and “changing into special clothing when entering hygiene control area” (81.6% (40/49)) was high. The farms where feed transportation services do not enter the hygiene control area were only 23.1% (12/52). The implementation rates of biosecurity measures in relation to veterinarians, i.e., “disinfecting veterinarians' vehicles,” “changing into special boots by veterinarians” and “cleaning veterinarians” hands before providing veterinary services,” were all ≥95.0%. The implementation rate of the biosecurity measures regarding the prevention of wildlife intrusion tended to be low; 66.0% (35/52) of farms had perimeter fences (30.2% (16/53) had electric fences), and 40.4% (19/47) and 37.0% (17/46) of farms had perimeter fences with a height of ≥1.5 m and mesh size of <5 cm, respectively.

The proportion of farms that implemented biosecurity measures set out in the new Biosecurity Standards introduced in 2020 in response to the current CSF epidemic was low. For example, “inspection of holes in pig house walls,” “mesh size of bird net ≤ 2 cm,” and “changing into special clothing for each pig house” were 21.6%, 44.7%, and 42.9%, respectively.

### 3.2. Results of Binomial Regression Analyses for the Phase in Which Infected Wild Boar Were Not Observed around Farms (Non-WB Phase)

The total number of weekly escapes of CSF outbreaks (total number of weeks observed for the 53 farms studied) during the non-WB phase was 1,864 in the prevalence approach and 1,821 in the TK approach, respectively. The number of farms where the virus was introduced (occurrences) during the study period was four in the prevalence approach and two in the TK approach. The number of farms where the virus was introduced during the study period was four in the prevalence approach and two in the TK approach. The differences between the two modeling approaches were attributed to the timing of WB/non-WB categorization (refer to Section 2.3 for details).  Table 3 shows variables with *p* < 0.2, as estimated by the Wald test in the univariable analysis (see full results in *Supplementary table 3*). Some variables were completely separated and could not be estimated. Using the prevalence approach, the following variables satisfied the condition: “no employment of foreign trainees,” “properly adjusting the concentration of disinfectant for vehicles,” “daily washing and disinfecting of clothing in pig houses,” “contract with veterinarians who provide hygiene guidance” and “installation of perimeter fences around the farm.” Consequently, in the TK approach, only “daily washing and disinfecting of clothing in pig houses” satisfied the condition.

Table 4 shows the results of multivariable analyses. There was no multicollinearity between variables in the present study. The final model in both the prevalence and TK approaches included one variable: “daily washing and disinfecting of clothing in pig houses” (coefficient = −2.28 (95% confidence interval (CI) = −4.40, −0.16) and −2.28 (95% CI = −5.52, 0.95), hazard ratio = 0.10 (95% CI = 0.01, 0.85) and 0.10 (95% CI = 0, 2.59), *p* = 0.023 and 0.107, respectively).

### 3.3. Results of Binomial Regressions for the Phase When Infected Wild Boar Were Observed around the Farm (WB Phase)

The total number of weekly escapes/occurrences of CSF outbreaks during the WB phase was 416 in the prevalence approach and 459 in the TK approach. The number of farms where the virus was introduced during the period was 17 in the prevalence approach and 19 in the TK approach.  Table 5 shows the result of the univariable analysis for variables with *p* < 0.2 in the Wald test (see all results in *Supplementary table 3*). The variables of “absence of public roads on farm,” “preventing wildlife intrusion into the areas used to store pig carcasses,” “no composting of pig carcasses on own farm,” “height of perimeter fence surrounding the farm ≥ 1.5 m” and “and mesh size of bird net ≤ 2 cm” satisfied the condition in both prevalence and TK approaches. The variables were subsequently used for the multivariable analyses.

The final model was identified as one with the lowest AIC among all combinations of these selected variables (Table 6). The prevalence model included “absence of public roads on the farm” (coefficient = −1.51 (95% CI = −2.77, −0.23), hazard ratio = 0.22 (95% CI = 0.06, 0.79), *p* = 0.019) and “preventing wildlife intrusion into areas used for the storage of pig carcasses” (coefficient = −1.03 (95% CI = −2.25, 0.33), hazard ratio = 0.36 (95% CI = 0.11, 1.39), *p* = 0.110) and “height of perimeter fence surrounding the farm ≥ 1.5 m” (coefficient = −1.22 (95% CI = −2.62, 0.38), hazard ratio = 0.30 (95% CI = 0.07, 1.46), *p* = 0.065). The TK model included “absence of roads in the farm” (coefficient = −1.11 (95% CI = −2.12, −0.05), hazard ratio = 0.33 ((95% CI = 0.12, 0.95), *p* = 0.034), “properly adjusting the concentration of disinfectant for vehicles” (coefficient = −1.16 (95% CI = −2.11, −0.10)), hazard ratio = 0.31 (95% CI = 0.12, 0.90), *p* = 0.023) and “preventing wildlife intrusion into areas used for the storage of pig carcasses” (coefficient = −1.13 (95% CI = −2.37, 0.13), hazard ratio = 0.32 (95% CI = 0.09, 1.14), *p* = 0.021).

## 4. Discussion

The present study evaluated the contribution of farm attributes and the effectiveness of biosecurity measures to prevent the introduction of CSFV onto farms from CSF-infected wild boar and farms. Two modeling approaches were used to assess wild boar-derived transmission risk, i.e., the prevalence approach and the transmission kernel-based (TK) approach. The analyses were conducted for two phases, i.e., where infected wild boar were/were not observed around farms (WB phase/non-WB phase).

In the multivariable analyses of the non-WB phase, only “daily washing and disinfecting of clothing in pig houses” was selected in both the prevalence and the TK approaches. In the analyses of the WB phase, “absence of public roads on the farm,” “preventing wildlife intrusion into areas used for the storage of pig carcasses,” and “height of perimeter fence surrounding the farm ≥ 1.5 m” were selected in the prevalence approach. In contrast, “absence of public roads on the farm,” “properly adjusting the concentration of disinfectant for vehicles,” and “preventing wildlife intrusion into areas used for the storage of pig carcasses” were selected in the TK approach.

In this study, farmers were asked about the farm attributes and the implementation status of biosecurity measures when a CSF outbreak was reported in the same prefecture as the farm. The implementation rates of biosecurity measures directed at preventing wildlife intrusion were low, and many farms were characterized as having conditions where small and medium-sized wild animals could easily enter the farm. After the inclusion of these measures in the Standards in 2020, current implementation rates of some measures have improved significantly (e.g., 94.4% in the case of installation of perimeter fences and 94.9% in the inspection of holes in the pig house walls in 2022) [43]. However, among the measures added in 2020, the adoption of some measures was found to be lacking (e.g., 75.2% for changing into special clothing and boots for each house in 2022) [43].

Caution is warranted when interpreting the effect of variables that were not considered or selected in the present study. The effect of biosecurity practices with extremely high implementation rates, such as biosecurity practices related to when veterinarians visit the farm, could not be evaluated in the present study. In addition, the effect of completely separated variables was not estimated, particularly in the non-WB phase of the TK approach, where data for only two outbreaks were available. It is important to emphasize that these findings did not demonstrate that the measures were not effective. Further, given the limited number of outbreak events, the estimates might be skewed by the chance effect. In fact, although the effects of the biosecurity practices such as “clarifying the border of hygienic control area” and “limiting access and disinfection at the farm entrance by visitors” were not significant in the present study, they have been suggested as effective measures against the introduction of ASF into farms elsewhere [6, 44]. Thus, to further minimize the risk of CSF outbreaks on farms, biosecurity measures that were not selected in the present study should nonetheless be implemented comprehensively on pig farms. Additionally, there would not be a single silver bullet to prevent CSF introduction. Given this background, an interpretation of the effect of selected biosecurity measures assessed in the present study is provided below.

In the non-WB phase, “daily washing and disinfecting of clothing in pig houses” was consistently selected in the prevalence and the TK approaches. It is widely known that cleaning and disinfecting contaminated work clothing are essential for preventing the mechanical transmission of pathogens [5, 6]. In the previous experimental study, the spread of CSFV was promoted by contaminated clothes and footwear [45]. The Biosecurity Standard requires the use of exclusive clothing and footwear and the cleaning and disinfection of contaminated clothing and footwear [18]. The result of this study will be used as adequate evidence to encourage farmers to implement these measures thoroughly.

In the presence of infected wild boar (i.e., the WB phase), “the absence of public roads in the farm” and “preventing wildlife intrusion into areas used for the storage of pig carcasses” were consistently selected by both the prevalence and the TK approaches. Additionally, in the TK approach, “properly adjusting the concentration of disinfectant for vehicles” was selected in the final model. The results suggest a less frequent contact with vehicles and an adequate vehicle disinfection reduce the risk of CSFV transmission into a farm. CSFV has been reported to survive for 4–5 days in feces at 20°C, and this means that contaminated transport vehicles can be a mechanical vector for the virus [8]. Since vehicles have also been reported as a risk factor for ASF outbreaks [46, 47] that might invade Japan, control measures against vehicles should be enhanced. The selection of “preventing wildlife intrusion into areas used for the storage of pig carcasses” highlights the effectiveness of implementing measures to prevent wildlife intrusion, particularly in locations within the farm where the risk of wildlife intrusion is high. As preventive measures, strategies such as weeding around the pig carcass storage area and conducting regular inspections of the storage facilities can be employed. In fact, the epidemiological studies on the infected farms, medium-sized animals (e.g., raccoon dogs and feral cats) and wild birds were observed on the infected farms [12, 13], and PCR tests of feces from cats and birds on the CSF-infected farms have been detected positive for CSFV [48]. These animals may have mechanically transmitted the virus into the farms. Therefore, areas that attract wildlife, including carcass storage areas, feedlots, and compost sheds [48], should be protected from contact with wild animals.

The present study has several limitations. First, the sample size of the present study was not large. This limitation is due to the dramatic decrease in the number of outbreaks on farms following the start of the vaccination campaign among farms at risk. The number of cases per month on pig farms before the start of the vaccination campaign (between September 2018 and December 2019) was 3.19, and that after the start of vaccination (between January 2020 and March 2023) was 0.90 [49]. Furthermore, some farms could not be included in the survey due to their closure after an outbreak. In the future, changes in the situation due to vaccination should be considered. Second, data on biosecurity practices on farms were assumed to be unchanged. The questions focused on the status of the biosecurity practices that were employed when the initial CSF case was reported in the prefecture where the respondent's farm was located. This was because the focus was on representing the status of the infected farms at the time of their infection. Third, Data for the wild boar population was not available. To address this problem, this study introduced two approaches: the prevalence approach, which does not use population data, and the transmission kernel-based approach, which assumes that the number of test-positive wild boar reflects the number of infected wild boar. The relationship between the population size of wild boar and the risk of infection should be modeled more comprehensively in the future and the population of wild boar should be estimated. Forth, data on the surveillance effort were also not available. The number of test-positives and detection timing might be biased by the difference in surveillance efforts between locations. Surveillance efforts should be measured and integrated for further analysis. Fifth, farm herd size, which could affect the implementation of biosecurity measures [50], was not considered in this study. This was because data on herd size on farms affected by CSF were unavailable. This should be considered as a factor in future research.

## 5. Conclusion

This study evaluated the contribution of farm attributes and the effectiveness of biosecurity measures on the prevention of CSF introduction in pig farms with and without infected wild boar present in the vicinity. On farms with no infected wild boar around, “daily washing and disinfecting of clothing in pig houses” was suggested as the measure to reduce the possibility of CSFV introduction into the farms. On farms with infected wild boar around, “absence of public roads on the farm” and “preventing wildlife intrusion into areas used for the storage of pig carcasses” were suggested to be effective. Of course, the comprehensive and strict implementation of other biosecurity measures are important; however, the implementation of those measures is worth being prioritized.

## Figures and Tables

**Table 1:** Contents of the questionnaire.

Categories	Items
(i) Farmers' attributes	Age and sex
(ii) Farm attributes	Farm address; management type; number of pigs; number of pig houses; number of staff
	*Adjacency to national or prefectural main roads*
	*Absence of public roads on the farm*
(iii) Biosecurity practices employed when farm staff enter and exit areas/facilities	—
Farm area	Parking staff's vehicles off site, or disinfecting them before entering the farm*⁣*^*∗*^^2^
	Properly adjusting the concentration of disinfectant for vehicles
	Separating entrances for employees'/feed transport vehicles and compost/pig transport vehicles
Hygienic control area	Clarifying the border of the hygienic control area*⁣*^*∗*^^2^
	Conducting showering-in
	Changing into special clothing*⁣*^*∗*^^2^
Pig houses	Daily washing and disinfecting of clothing in pig houses
	Changing into special clothing for each pig house
	Changing boots for each pig house
	Establishing clear zones for where to place outer and inner boots
	Disinfecting boots in a disinfection bath
	Daily replacement of disinfectant in the boot bath
	Properly adjusting the concentration of disinfectants for boot disinfection
	Cleaning the dirt off boots before stepping into the disinfectant bath
	Disinfecting hands or wearing hygienic gloves*⁣*^*∗*^^2^
(iv) Biosecurity practices for rearing pigs	Assignment of personnel for each pig development stage
	Moving pigs by having them walk on the ground
(v) Biosecurity practices for feed and drinking water for pigs	No use of mountain stream water for drinking water
	No use of human food waste from meat processing facilities as feed
(vi) Biosecurity practice regarding farm staff	Absence of staff living on the farm premises
	No employment of foreign trainees
	No attending social gatherings with other pig farmers
(vii) Biosecurity practices when the farm was visited by persons other than farm employees	—
Feed transport services	Restricting entry of feed transport vehicles into the hygienic control area
Veterinarian services	Contract with veterinarians who provide hygiene guidance*⁣*^*∗*^^2^
	Disinfecting veterinarians' vehicles*⁣*^*∗*^^2^
	Changing into special boots*⁣*^*∗*^^1^*⁣*^*∗*^^2^
	Cleaning hands before providing veterinary services*⁣*^*∗*^^1^*⁣*^*∗*^^2^
Facility construction services	Disinfecting construction tools transported into the farm*⁣*^*∗*^^2^
	Changing into special clothing and boots*⁣*^*∗*^^2^
	Disinfecting hands*⁣*^*∗*^^2^
Other outside visitors	Restricting the entry of visitors not related to the farm*⁣*^*∗*^^2^
	Restricting the entry of hunters*⁣*^*∗*^^1^*⁣*^*∗*^^3^
(viii) Biosecurity practices to prevent intrusion of wildlife	—
Management of disposed materials	No use of a common compost station
	Preventing wildlife intrusion into areas used for the storage of pig carcasses*⁣*^*∗*^^2^
	No composting of pig carcasses on own farm
	Contract with service for disposal of pig carcasses
Perimeter fences	Installation of perimeter fences around the farm
	Height of perimeter fence surrounding the farm ≥ 1.5 m
	Mesh size of perimeter fence surrounding the farm < 5 cm
	Closing the entrance of perimeter fence
Control of small wild animals	Inspection of holes in pig house walls
	Preventing the intrusion of mice and other small wild animals in pig houses*⁣*^*∗*^^2^
Control of medium-sized wild animals	Preventing the intrusion of feral cats, raccoons, and other medium-sized wild animals in the hygienic control area
	Preventing the intrusion of feral cats, raccoons, and other medium-sized wild animals in pig houses
Control of wild birds	Installation of bird nets around pig houses
	Mesh size of bird net ≤2 cm
	Preventing the intrusion of wild birds in pig houses

Forty-seven items, including those italic in “Farm attributes” and all biosecurity practices, were used as explanatory variables in the analysis; *⁣*^*∗*^^1^, variables excluded from the final analyses because of their extremely high implementation rates; *⁣*^*∗*^^2^, the items in the Standard Biosecurity as of 2018; *⁣*^*∗*^^3^, hunters can be visitors to the farm, or farm staff can be hunters.

**Table 2:** Response rate of the questionnaire.

Prefectures	Infected farms	Noninfected farms	Total
Aichi	27.5% (11/45)	40.0% (26/65)	35.2% (37/105)
Fukui	—	0.0% (0/2)	0.0% (0/2)
Gifu	83.3% (15/18)	44.4% (4/9)	70.4% (19/27)
Mie	100.0% (1/1)	—	100.0% (1/1)
Nagano	100.0% (1/1)	25.0% (2/8)	33.3% (3/9)
Yamanashi	0.0% (0/1)	50.0% (1/2)	33.3% (1/3)
Total	42.4% (28/66)	38.4% (33/86)	40.1% (61/152)

**Table 3:** Results of univariable analyses using the prevalence approach and the transmission kernel (TK) approach during the phase when infected wild boar were not observed around the farms (non-WB phase).

Selected variables	Prevalence approach			TK approach		
	Coefficient (95% CI)	Hazard ratio (95% CI)	*p*-Value	Coefficient (95% CI)	Hazard ratio (95% CI)	*p*-Value
No employment of foreign trainees	−1.36 (−3.49, 0.78)	0.26 (0.03, 2.18)	0.178	NA^a^	NA^a^	NA^a^

Properly adjusting the concentration of disinfectant for vehicles	−1.83 (−3.95, 0.30)	0.16 (0.02, 1.35)	0.068	NA^a^	NA^a^	NA^a^

Daily washing and disinfecting of clothing in pig houses	−2.28 (−4.40, −0.16)	0.10 (0.01, 0.85)	0.026*⁣*^*∗*^	−2.28 (−5.52, 0.95)	0.10 (0, 2.59)	0.107

Contract with veterinarians who provide hygiene guidance	−1.60 (−3.74, 1.43)	0.20 (0.02, 4.18)	0.175	NA^a^	NA^a^	NA^a^

Installation of perimeter fences around the farm	−1.62 (−4.00, 0.59)	0.20 (0.02, 1.80)	0.131	NA^a^	NA^a^	NA^a^

Only variables with *p* < 0.20 are shown here; for simplicity, the coefficient of intercept and the transmission risk from infected farms for each univariable analysis are not shown; *⁣*^*∗*^*p* < 0.05; ^a^NA, not estimated due to complete separation.

**Table 4:** Results of multivariable analyses in the prevalence and transmission kernel (TK) approached phase when infected wild boars were not observed around the farm (non-WB phase).

Variables	Prevalence approach			TK approach		
	Coefficient (95% CI)	Hazard ratio (95% CI)	*p*-Value	Coefficient (95% CI)	Hazard ratio (95% CI)	*p*-Value
Intercept	−4.64 (−6.53, −3.42)	0.01 (0, 0.03)	<0.001*⁣*^*∗*^	−5.56 (−8.67, −3.82)	0 (0, 0.02)	<0.001*⁣*^*∗*^

Transmission risk from infected farms	0.72 (−0.77, 1.53)	2.05 (0.46, 4.62)	0.162	1.08 (−0.40, 2.07)	2.94 (0.67, 7.92)	0.048*⁣*^*∗*^

Daily washing and disinfecting of clothing in pig houses	−2.28 (−4.40, −0.16)	0.10 (0.01, 0.85)	0.023*⁣*^*∗*^	−2.28 (−5.52, 0.95)	0.10 (0, 2.59)	0.107

*⁣* ^*∗*^ *p* < 0.05.

**Table 5:** Results of univariable analyses using the prevalence and transmission kernel (TK) approaches during the phase when infected wild boar were observed around the farms (WB phase).

Selected variables	Prevalence approach			TK approach		
	Coefficient (95% CI)	Hazard ratio (95% CI)	*p*-Value	Coefficient (95% CI)	Hazard ratio (95% CI)	*p*-Value
Absence of public roads in the farm	−0.73 (−1.77, 0.40)	0.48 (0.17, 1.49)	0.181	−0.69 (−1.68, 0.35)	0.50 (0.19, 1.42)	0.175
Properly adjusting the concentration of disinfectant for vehicles	−0.45 (−1.51, 0.83)	0.64 (0.22, 2.29)	0.440	−0.88 (−1.81, 0.17)	0.41 (0.16, 0.84)	0.076
No entering of feed transport vehicles into the hygienic control area	−0.66 (−2.14, 0.49)	0.52 (0.12, 1.63)	0.311	−0.89 (−2.37, 0.26)	0.41 (0.09, 1.30)	0.168
Preventing wildlife intrusion into areas used for the storage of pig carcasses	−0.95 (−2.04, 0.35)	0.39 (0.13, 1.42)	0.106	−1.00 (−2.03, 0.18)	0.37 (0.13, 1.20)	0.069
No composting of pig carcasses on own farm	−0.65 (−1.61, 0.34)	0.52 (0.20, 1.40)	0.186	−0.74 (−1.71, 0.23)	0.48 (0.18, 1.26)	0.127
Height of perimeter fence surrounding the farm ≥ 1.5 m	−0.83 (−2.16, 0.43)	0.44 (0.12, 1.54)	0.156	−0.71 (−2.16, 0.43)	0.49 (0.12, 1.54)	0.176
Mesh size of perimeter fence surrounding the farm < 5 cm	−0.69 (−2.13, 0.25)	0.50 (0.12, 1.28)	0.280	−0.94 (−1.86, 0.26)	0.39 (0.16, 1.30)	0.137
Mesh size of bird net ≤ 2 cm	−0.69 (−1.71, 0.37)	0.50 (0.18, 1.45)	0.187	−0.83 (−1.81, 0.14)	0.44 (0.16, 1.15)	0.088

Only variables with *p* < 0.20 are shown here; for simplicity, the coefficient of intercept and the transmission risk from infected farms for each univariable analysis are not shown; *⁣*^*∗*^*p* < 0.05.

**Table 6:** Results of multivariable analyses using the prevalence and transmission kernel (TK) approaches during the phase when infected wild boar were observed around the farm (WB phase).

Variables	Prevalence approach			TK approach		
	Coefficient (95% CI)	Hazard ratio (95% CI)	*p*-Value	Coefficient (95% CI)	Hazard ratio (95% CI)	*p*-Value
Intercept	−1.29 (−3.11, 0.38)	0.28 (0.04, 1.46)	0.143	−0.79 (−2.24, 0.27)	0.45 (0.11, 1.31)	0.228
Transmission risk from infected farms	0.14 (−1.73, 1.46)	1.15 (0.18, 4.31)	0.853	0.22 (−1.64, 1.49)	1.25 (0.19, 4.44)	0.776
Transmission risk from infected wild boar	1.06 (−0.82, 2.90)	2.89 (0.44, 18.17)	0.258	0.37 (−0.07, 0.78)	1.45 (0.93, 2.18)	0.074
Absence of public roads in the farm	−1.51 (−2.77, −0.23)	0.22 (0.06, 0.79)	0.019*⁣*^*∗*^	−1.11 (−2.12, −0.05)	0.33 (0.12, 0.95)	0.034*⁣*^*∗*^
Properly adjusting the concentration of disinfectant for vehicles	—	—	—	−1.16 (−2.11, −0.10)	0.31 (0.12, 0.90)	0.023*⁣*^*∗*^
Preventing wildlife intrusion into areas used for the storage of pig carcasses	−1.03 (−2.25, 0.33)	0.36 (0.11, 1.39)	0.110	−1.13 (−2.37, 0.13)	0.32 (0.09, 1.14)	0.021*⁣*^*∗*^
Height of perimeter fence surrounding the farm ≥ 1.5 m	−1.22 (−2.62, 0.38)	0.30 (0.07, 1.46)	0.065	—	—	—

*⁣* ^*∗*^ *p* < 0.05.

## Data Availability

The data that support the findings of this study are not publicly available as they partially contain information that could compromise the privacy of research participants. The data can be considered for availability from the authors upon reasonable request.

## Supplementary Materials

Supplementary 1 Figure S1: weekly prevalence of CSF among wild boar within a 10-km radius of each farm.

Supplementary 2 Figure S2: a 3-week centered moving average of weekly prevalence of CSF among wild boar within a 10-km radius of each farm.

Supplementary 3 Table S1: proportions of farms that meet the farm attributes and implementation rates of biosecurity measures. Table S2: results of univariable analyses using the prevalence and transmission kernel (TK) approaches during the non-wild boar (non-WB) phase. “NA” denotes a variable that was not estimated due to complete separation. Table S3: results of univariable analyses using the prevalence and transmission kernel (TK) approaches during the wild boar (WB) phase. “NA” denotes a variable that was not estimated due to complete separation.
