# Supplementary material for: Identifying Effective Biosecurity Measures for Preventing the Introduction of Classical Swine Fever in Pig Farms in Japan: Under the Condition of Absence/Presence of Observable Infected Wild Boar
Source: Transbound Emerg Dis. 2024 Jul 31;2024:1305664. doi: 10.1155/2024/1305664 (PMC12017048; doi:10.1155/2024/1305664)

Farm 01

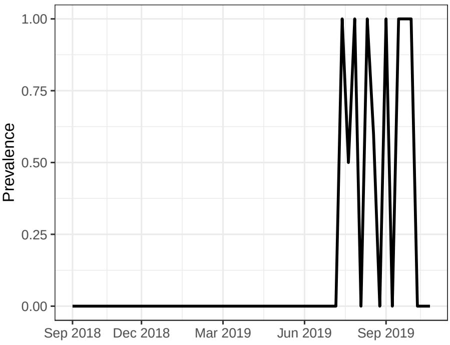

Farm 02

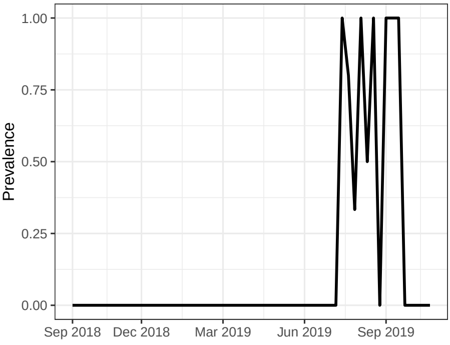

Farm 03

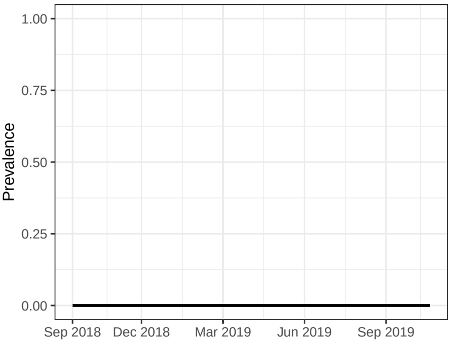

Farm 04

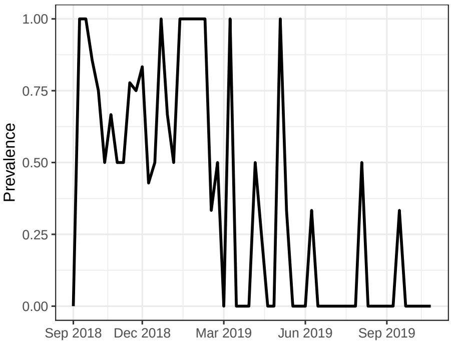

Farm 05

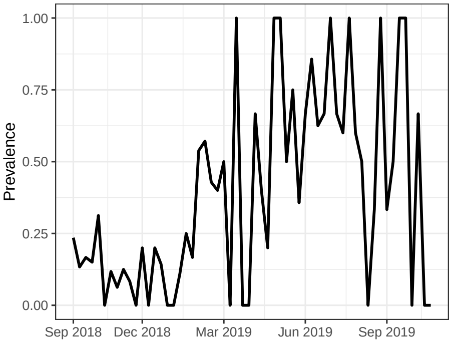

Farm 06

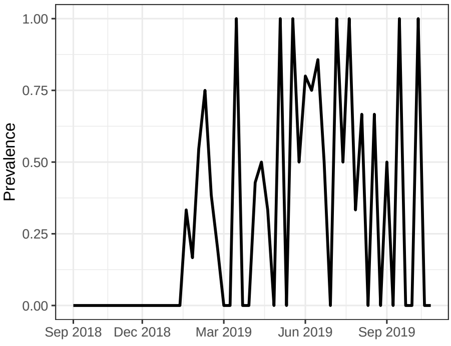

Farm 07

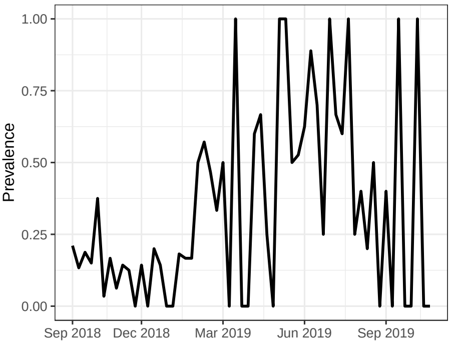

Farm 10

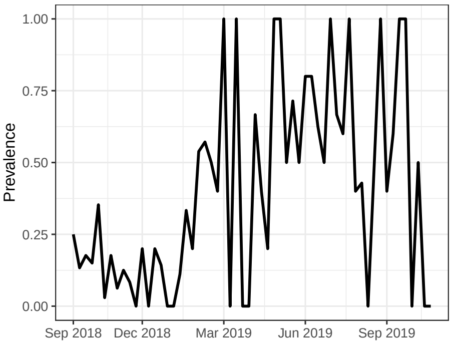

Farm 08

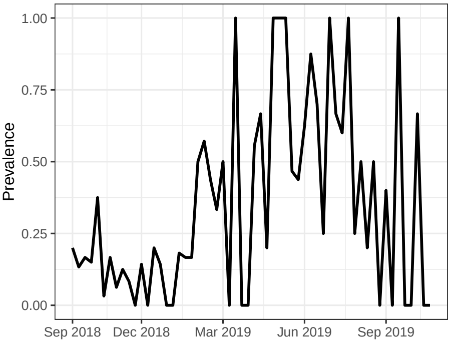

Farm 11

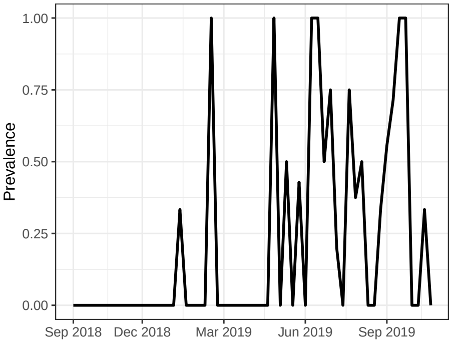

Farm 09

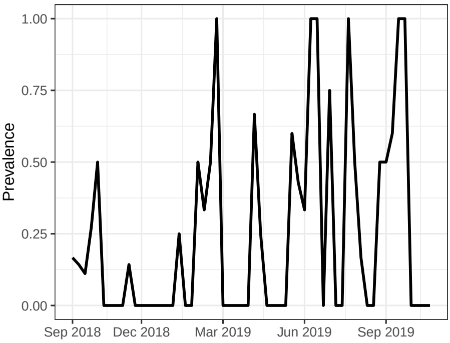

Farm 12

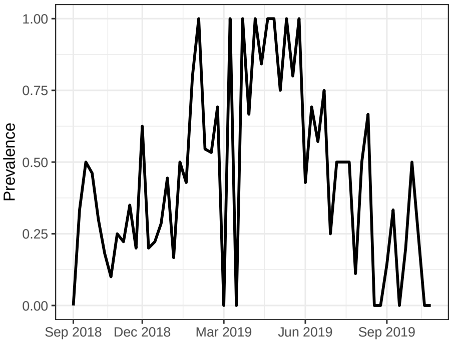

Farm 13

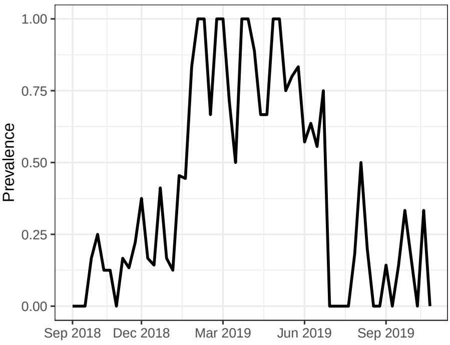

Farm 16

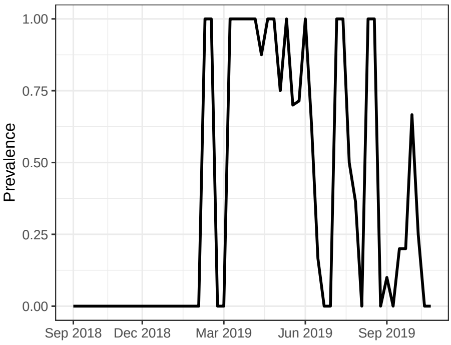

Farm 14

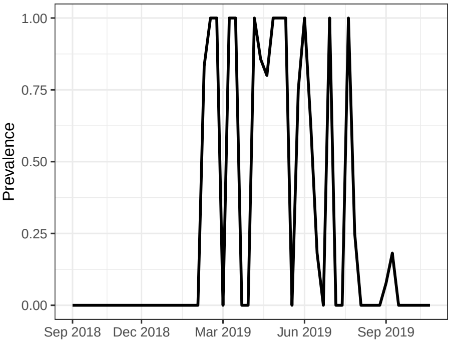

Farm 17

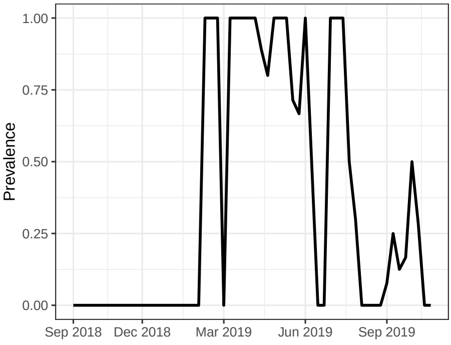

Farm 15

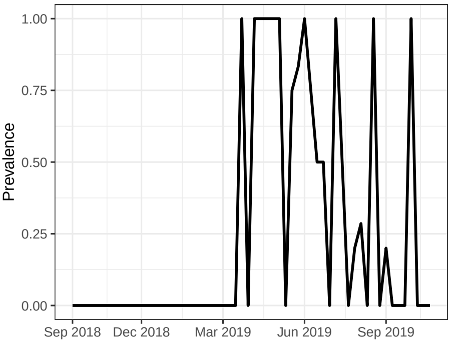

Farm 18

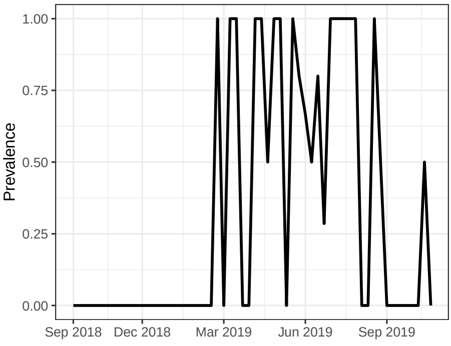

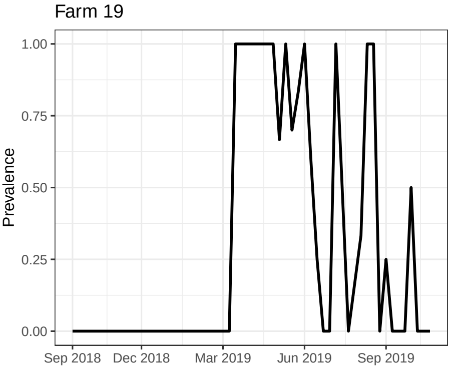
Farm 19
Prevalence
1.00
0.75
0.50
0.25
0.00
Sep 2018
Dec 2018
Mar 2019
Jun 2019
Sep 2019

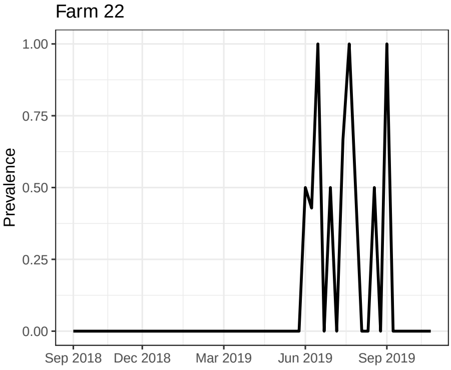
Farm 22
Prevalence
1.00
0.75
0.50
0.25
0.00
Sep 2018
Dec 2018
Mar 2019
Jun 2019
Sep 2019

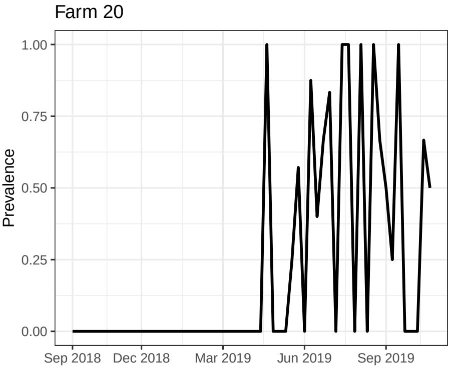
Farm 20
Prevalence
1.00
0.75
0.50
0.25
0.00
Sep 2018
Dec 2018
Mar 2019
Jun 2019
Sep 2019

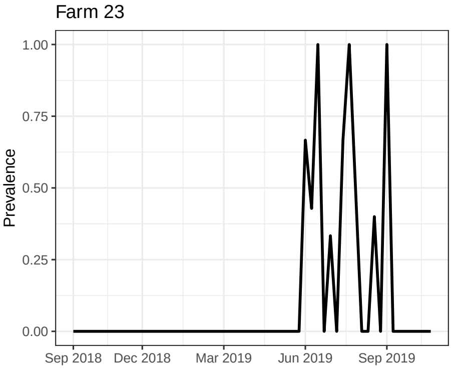
Farm 23
Prevalence
1.00
0.75
0.50
0.25
0.00
Sep 2018
Dec 2018
Mar 2019
Jun 2019
Sep 2019

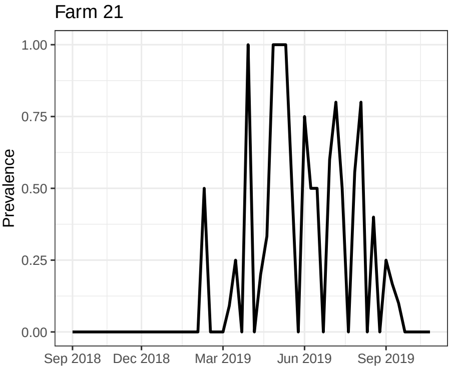
Farm 21
Prevalence
1.00
0.75
0.50
0.25
0.00
Sep 2018
Dec 2018
Mar 2019
Jun 2019
Sep 2019

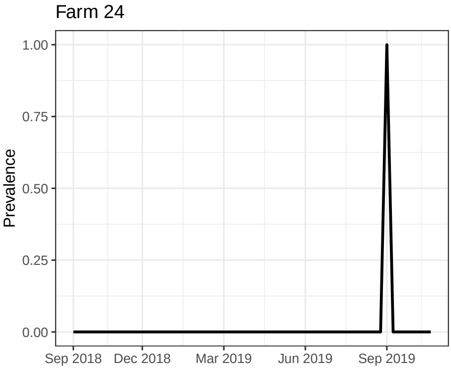
Farm 24
Prevalence
1.00
0.75
0.50
0.25
0.00
Sep 2018
Dec 2018
Mar 2019
Jun 2019
Sep 2019

Farm 25

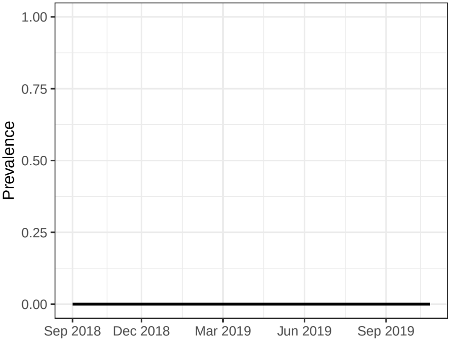

Farm 26

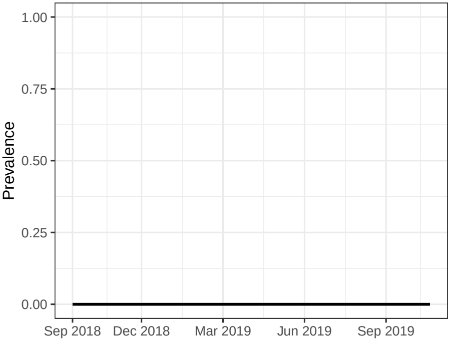

Farm 27

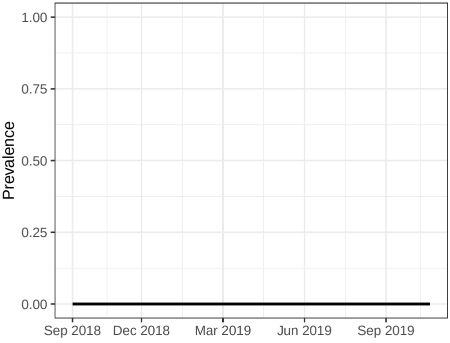

Farm 28

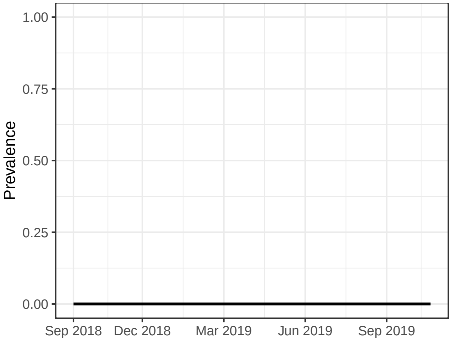

Farm 29

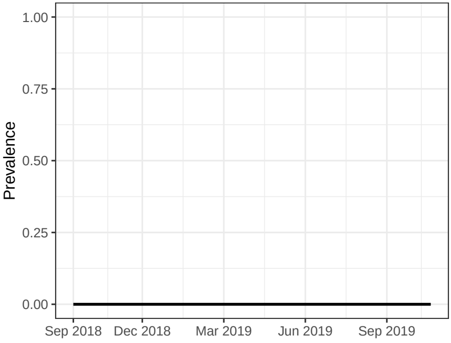

Farm 30

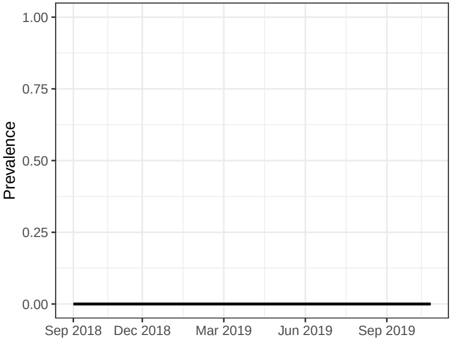

Farm 31

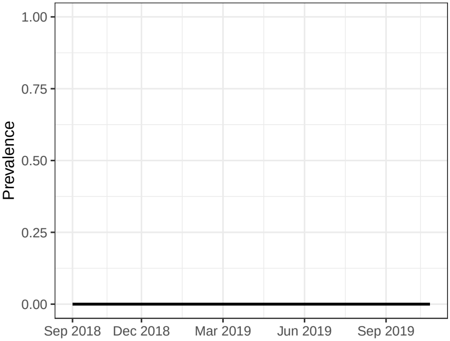

Farm 32

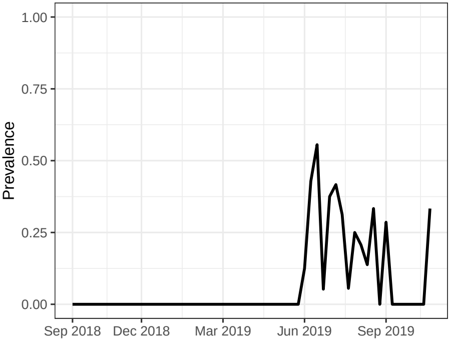

Farm 33

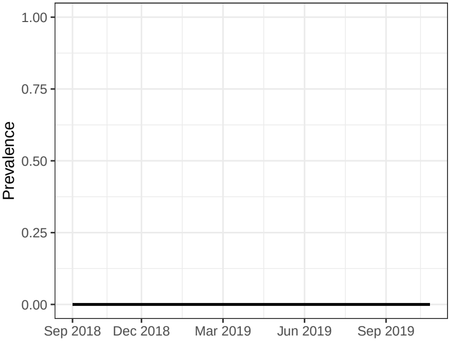

Farm 34

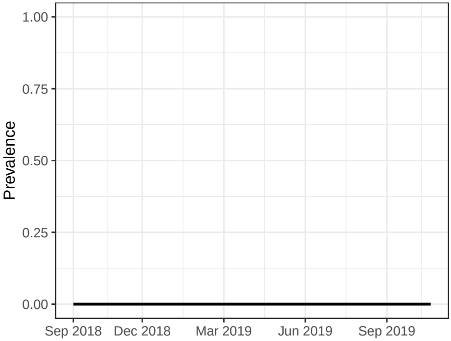

Farm 35

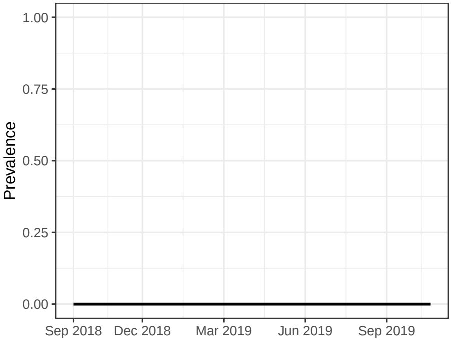

Farm 36

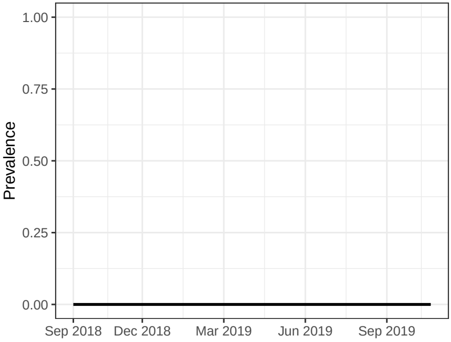

Farm 37

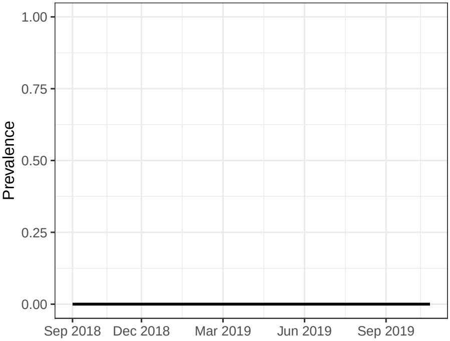

Farm 40

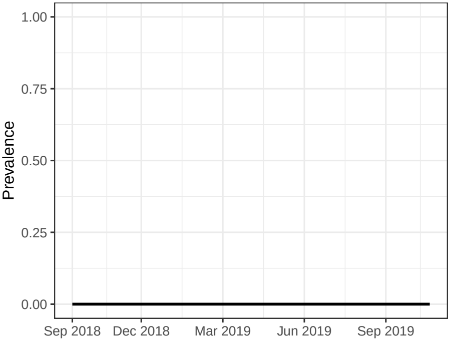

Farm 38

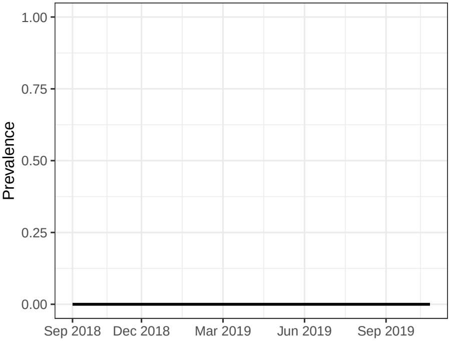

Farm 41

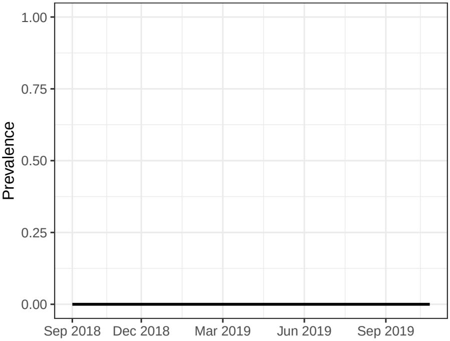

Farm 39

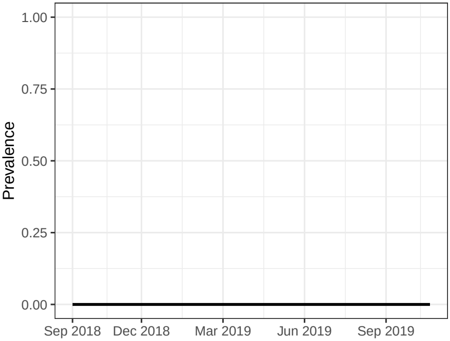

Farm 42

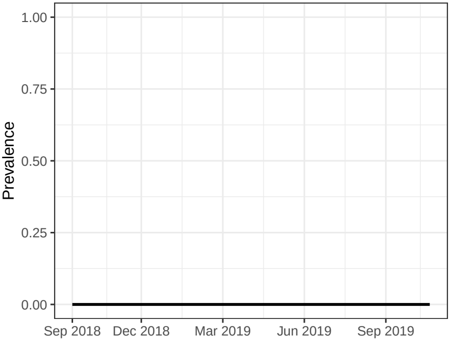

Farm 43

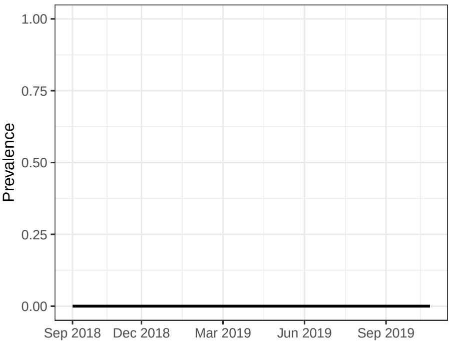

Farm 44

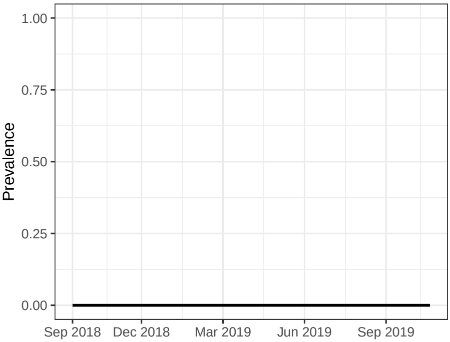

Farm 45

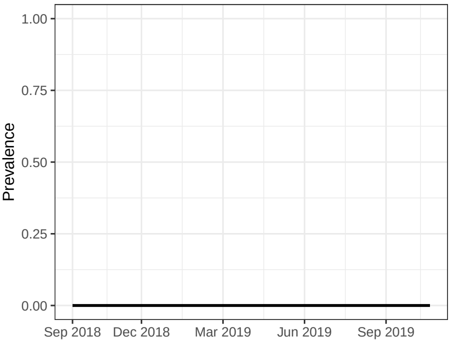

Farm 46

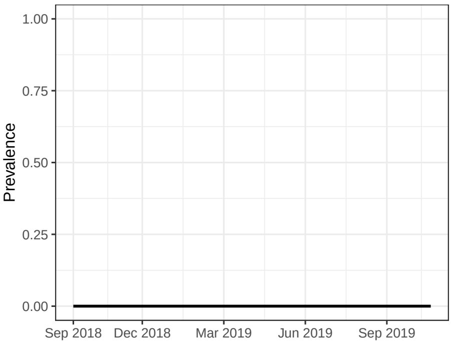

Farm 47

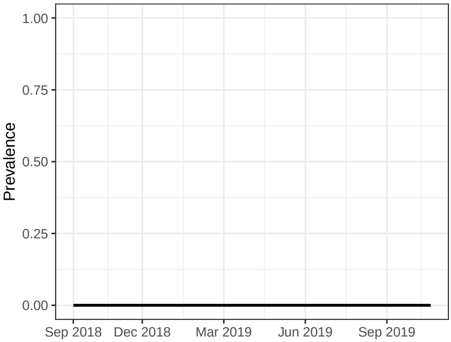

Farm 48

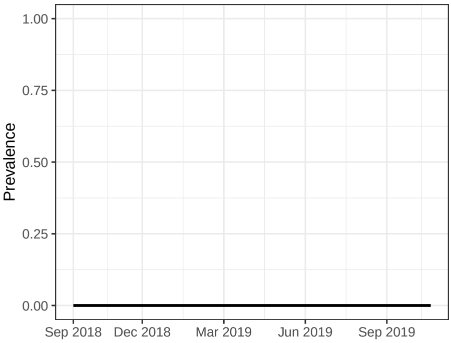

Farm 49

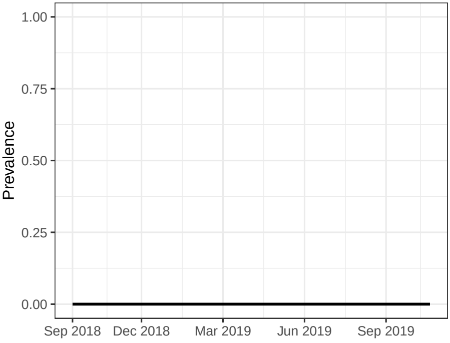

Farm 52

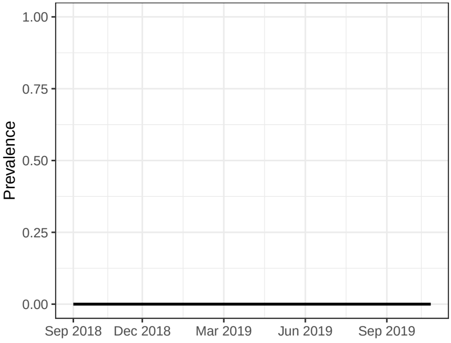

Farm 50

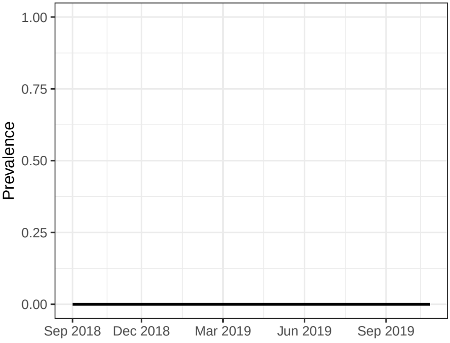

Farm 53

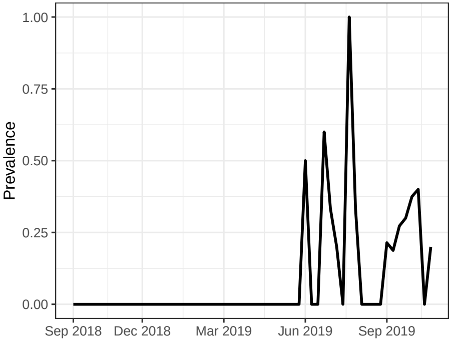

Farm 51

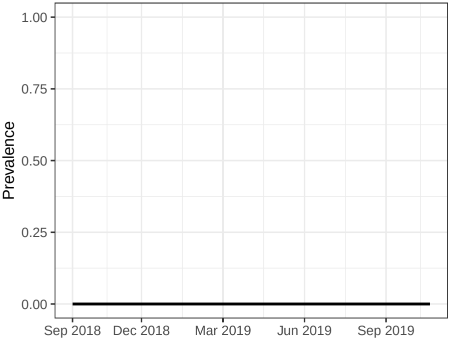

Supplement: Supplementary 1 — Figure S1: weekly prevalence of CSF among wild boar within a 10-km radius of each farm. [file 1305664.f1.pdf]
