# Supplementary material for: Identifying Effective Biosecurity Measures for Preventing the Introduction of Classical Swine Fever in Pig Farms in Japan: Under the Condition of Absence/Presence of Observable Infected Wild Boar
Source: Transbound Emerg Dis. 2024 Jul 31;2024:1305664. doi: 10.1155/2024/1305664 (PMC12017048; doi:10.1155/2024/1305664)

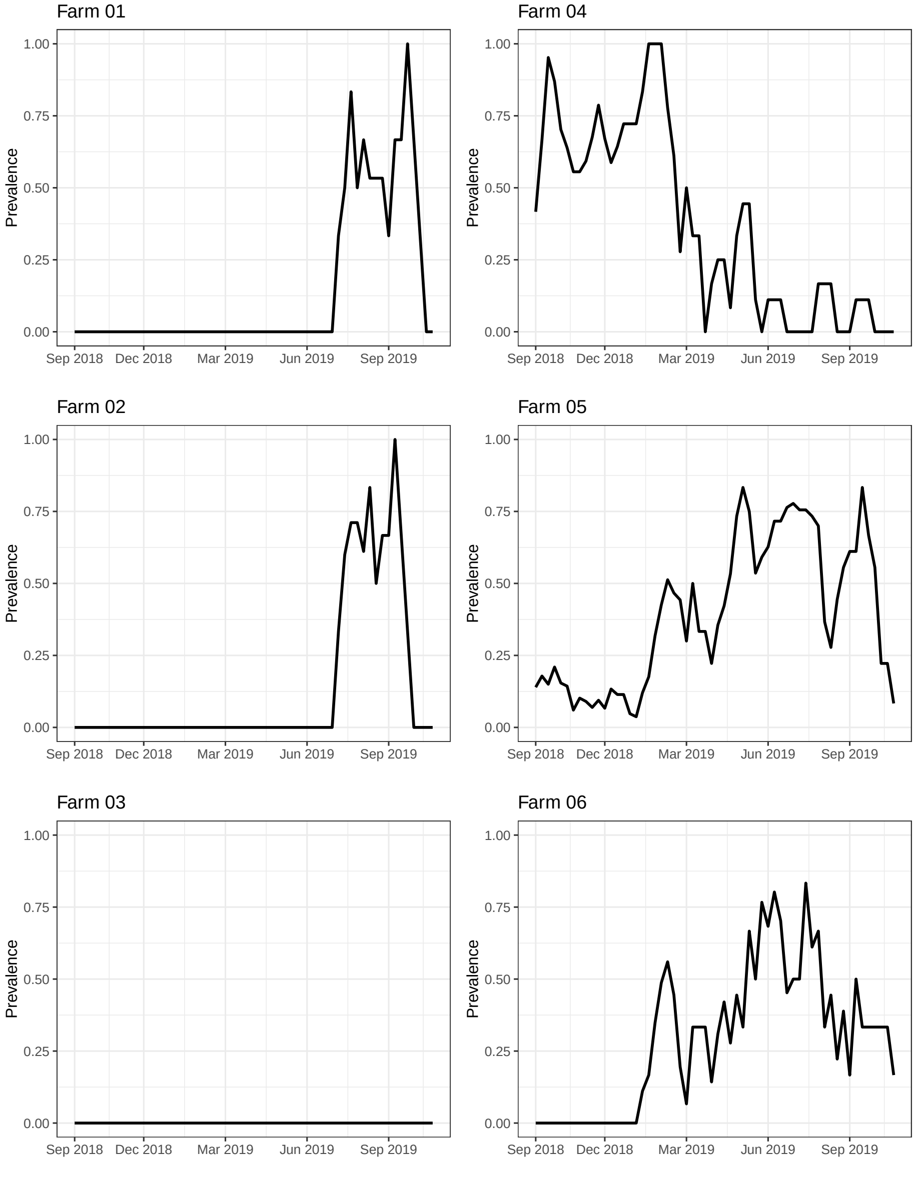
Farm 01
Farm 04
Farm 02
Farm 05
Farm 03
Farm 06
Prevalence
1.00
0.75
0.50
0.25
0.00
Sep 2018
Dec 2018
Mar 2019
Jun 2019
Sep 2019

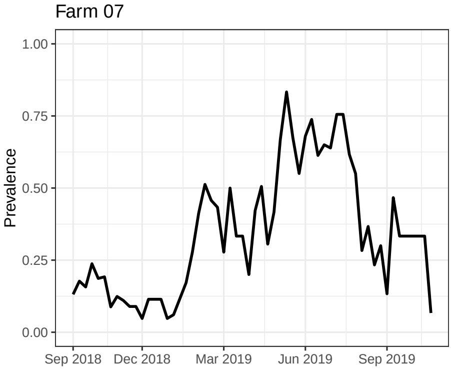
Farm 07
Prevalence
1.00
0.75
0.50
0.25
0.00
Sep 2018
Dec 2018
Mar 2019
Jun 2019
Sep 2019

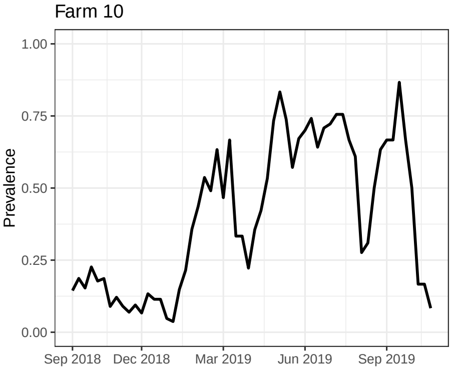
Farm 10
Prevalence
1.00
0.75
0.50
0.25
0.00
Sep 2018
Dec 2018
Mar 2019
Jun 2019
Sep 2019

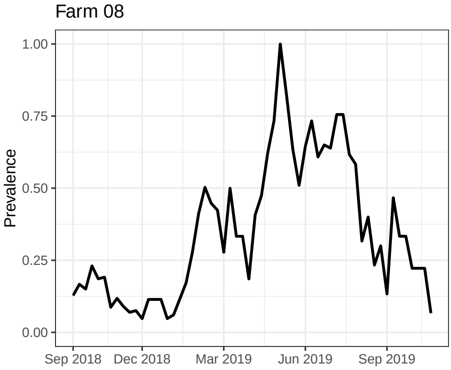
Farm 08
Prevalence
1.00
0.75
0.50
0.25
0.00
Sep 2018
Dec 2018
Mar 2019
Jun 2019
Sep 2019

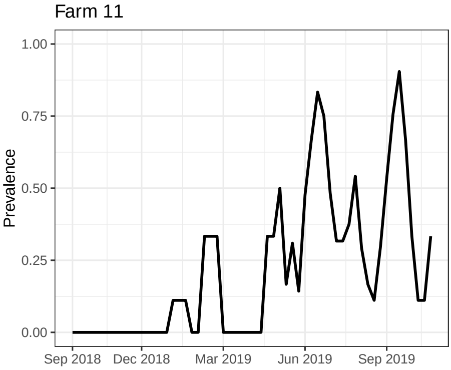
Farm 11
Prevalence
1.00
0.75
0.50
0.25
0.00
Sep 2018
Dec 2018
Mar 2019
Jun 2019
Sep 2019

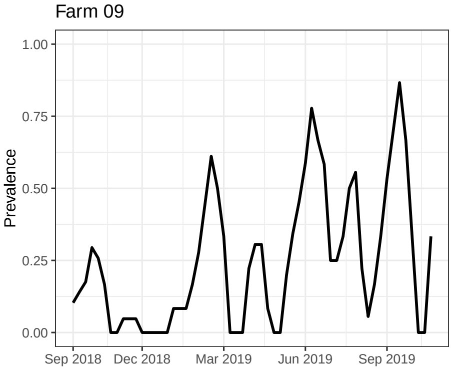
Farm 09
Prevalence
1.00
0.75
0.50
0.25
0.00
Sep 2018
Dec 2018
Mar 2019
Jun 2019
Sep 2019

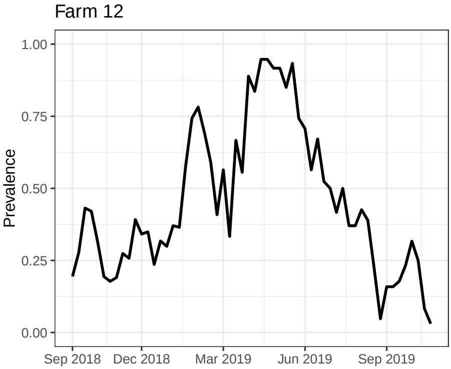
Farm 12
Prevalence
1.00
0.75
0.50
0.25
0.00
Sep 2018
Dec 2018
Mar 2019
Jun 2019
Sep 2019

Farm 13

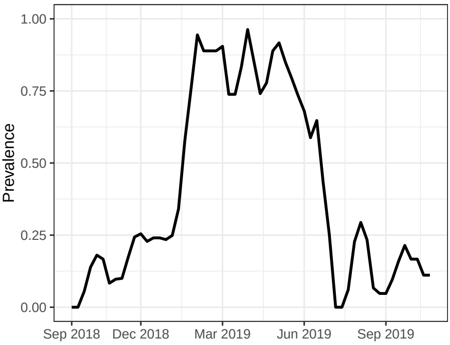

Farm 16

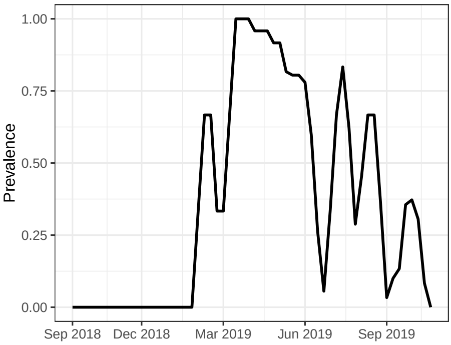

Farm 14

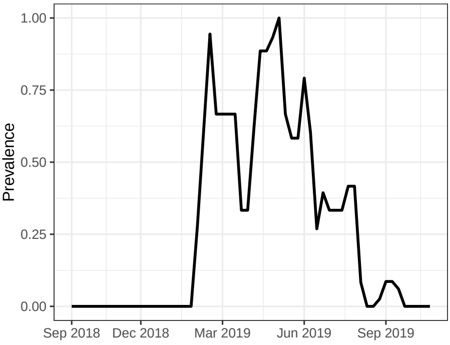

Farm 17

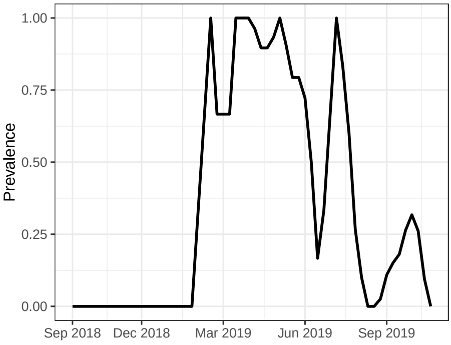

Farm 15

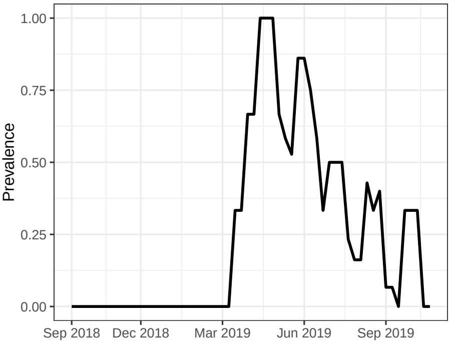

Farm 18

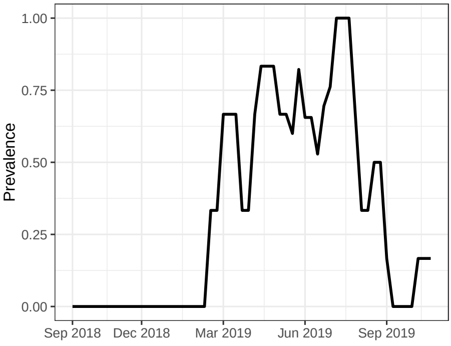

Farm 19

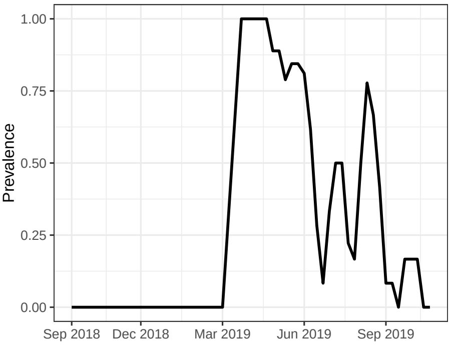

Farm 20

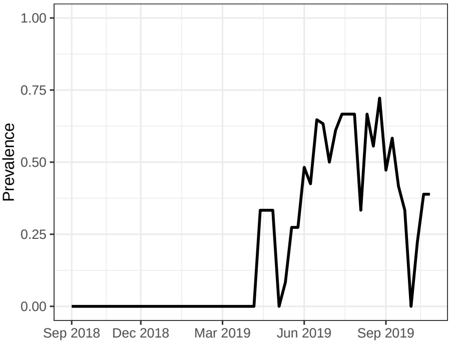

Farm 21

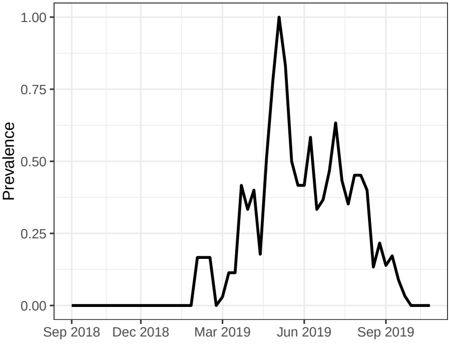

Farm 22

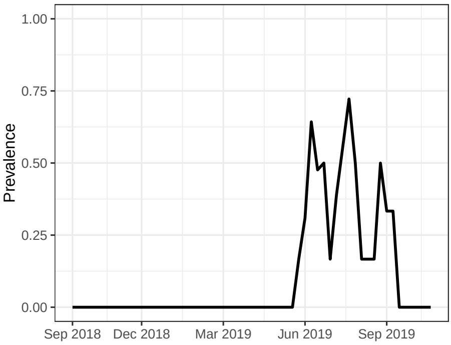

Farm 23

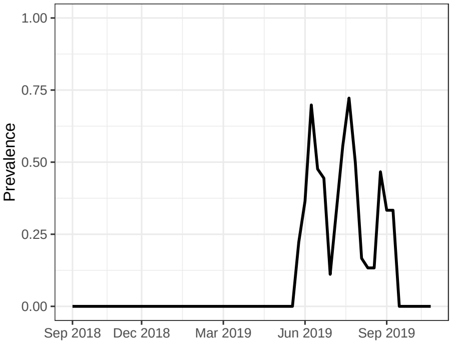

Farm 24

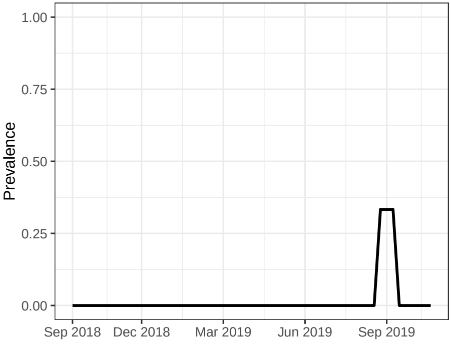

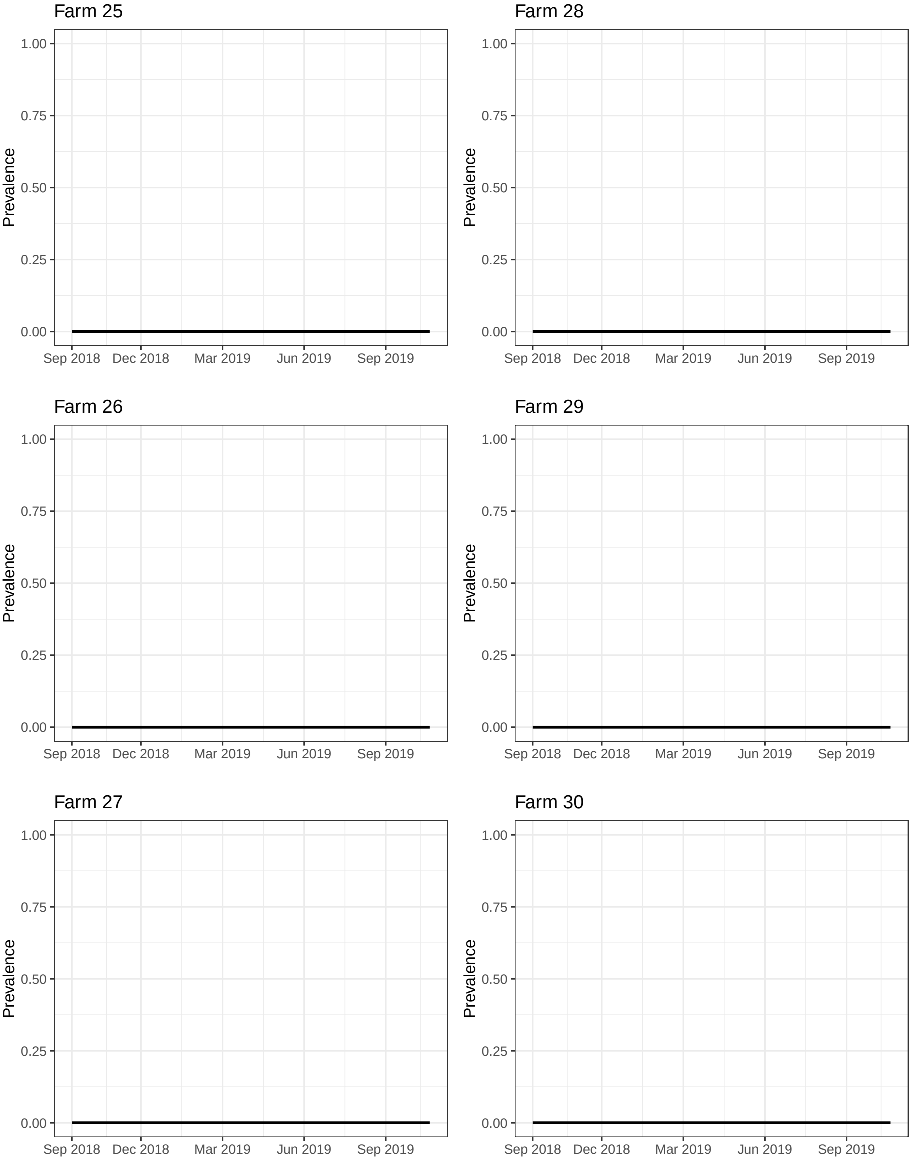
Farm 25
Farm 28
Farm 26
Farm 29
Farm 27
Farm 30
Prevalence
1.00
0.75
0.50
0.25
0.00
Sep 2018
Dec 2018
Mar 2019
Jun 2019
Sep 2019

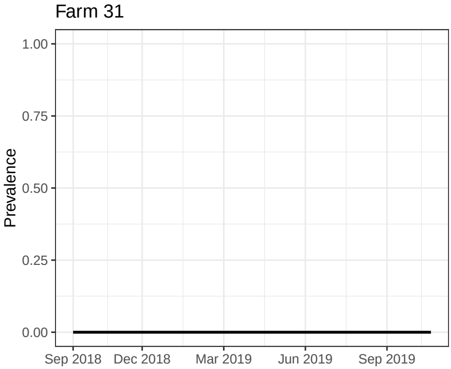
Farm 31
Prevalence
1.00
0.75
0.50
0.25
0.00
Sep 2018
Dec 2018
Mar 2019
Jun 2019
Sep 2019

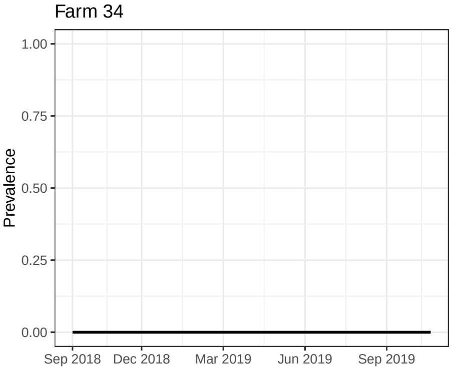
Farm 34
Prevalence
1.00
0.75
0.50
0.25
0.00
Sep 2018
Dec 2018
Mar 2019
Jun 2019
Sep 2019

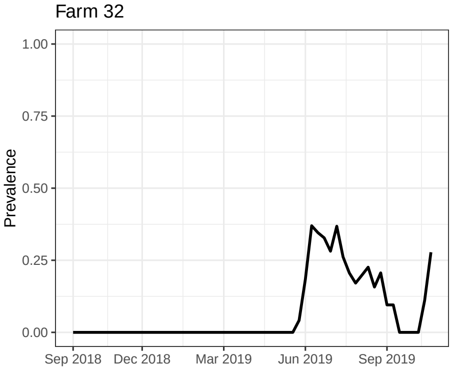
Farm 32
Prevalence
1.00
0.75
0.50
0.25
0.00
Sep 2018
Dec 2018
Mar 2019
Jun 2019
Sep 2019

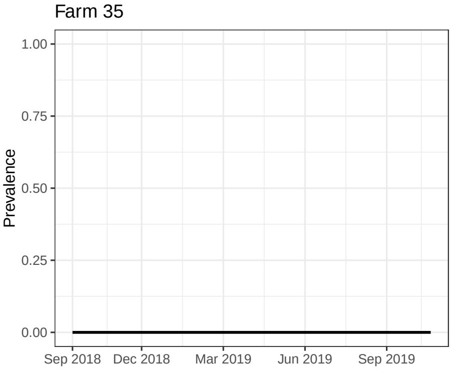
Farm 35
Prevalence
1.00
0.75
0.50
0.25
0.00
Sep 2018
Dec 2018
Mar 2019
Jun 2019
Sep 2019

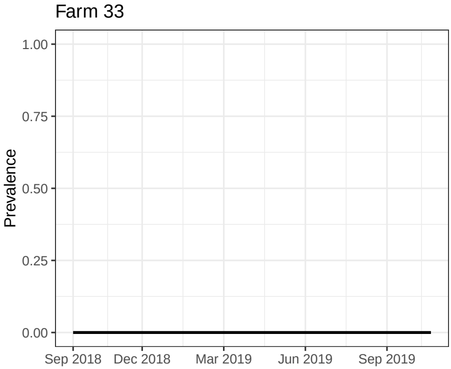
Farm 33
Prevalence
1.00
0.75
0.50
0.25
0.00
Sep 2018
Dec 2018
Mar 2019
Jun 2019
Sep 2019

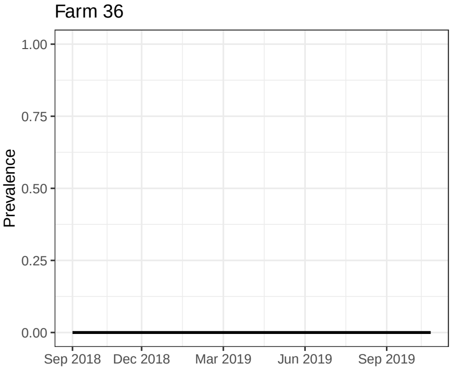
Farm 36
Prevalence
1.00
0.75
0.50
0.25
0.00
Sep 2018
Dec 2018
Mar 2019
Jun 2019
Sep 2019

Farm 37

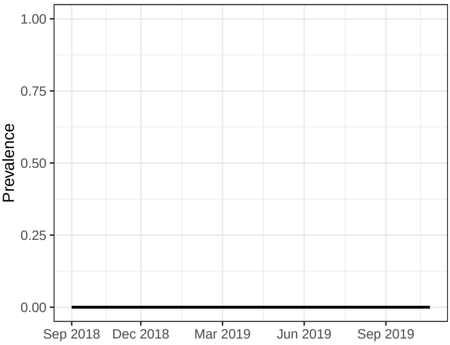

Farm 40

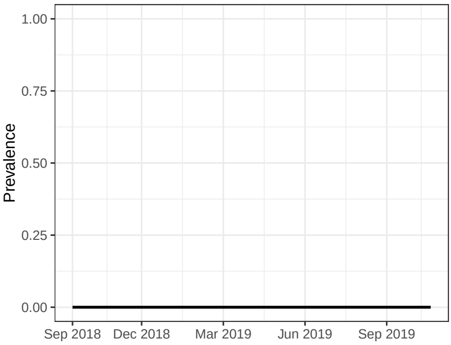

Farm 38

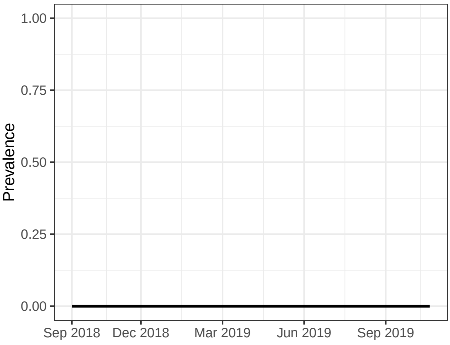

Farm 41

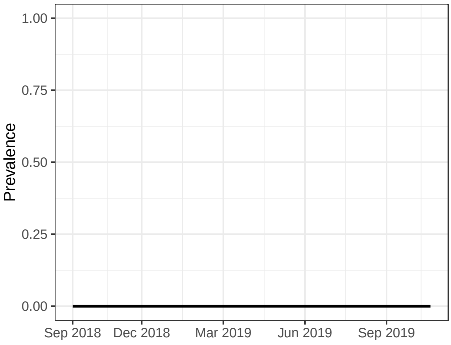

Farm 39

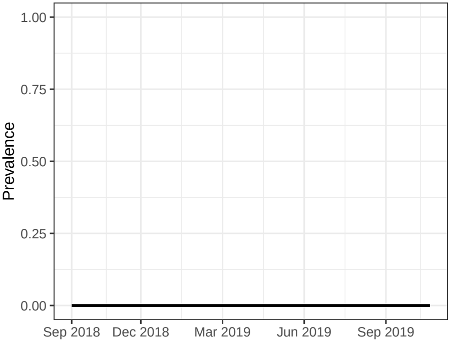

Farm 42

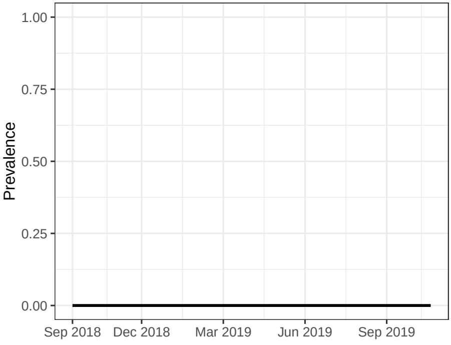

Farm 43

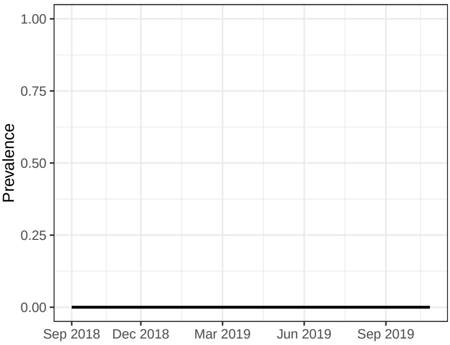

Farm 44

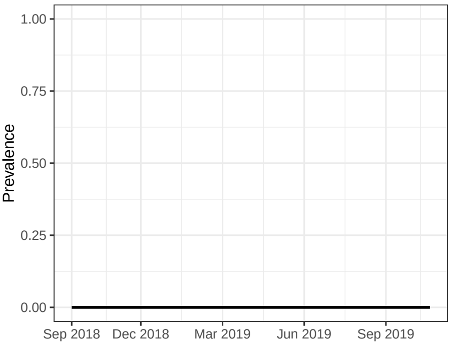

Farm 45

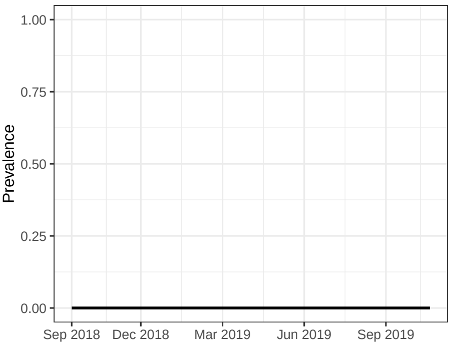

Farm 46

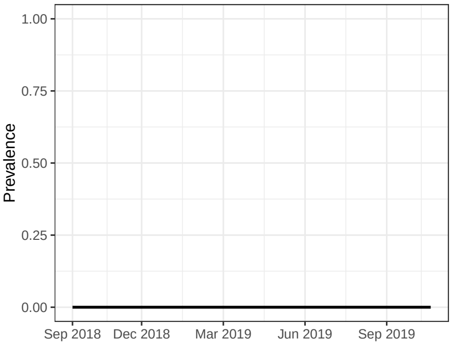

Farm 47

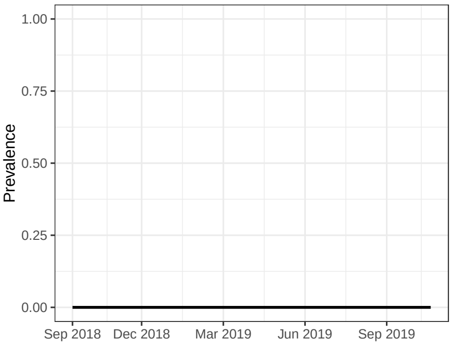

Farm 48

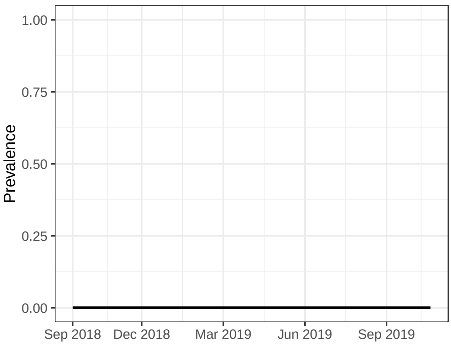

Farm 49

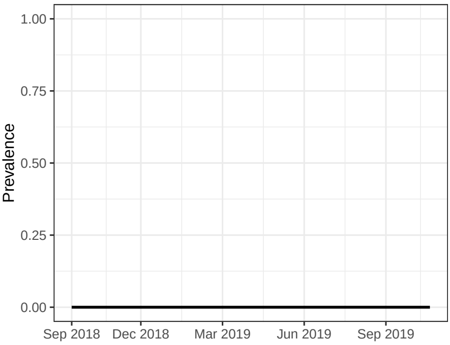

Farm 52

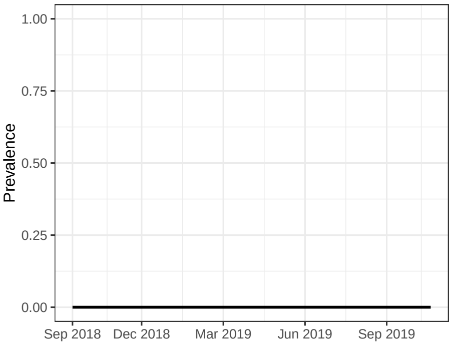

Farm 50

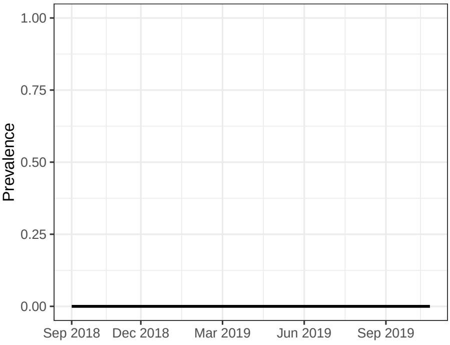

Farm 53

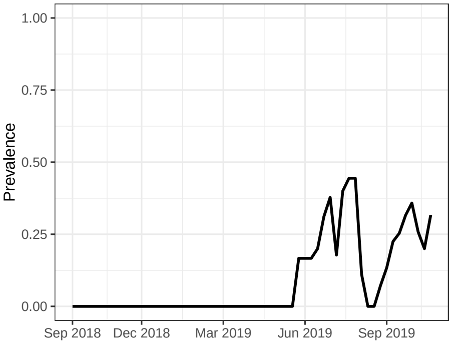

Farm 51

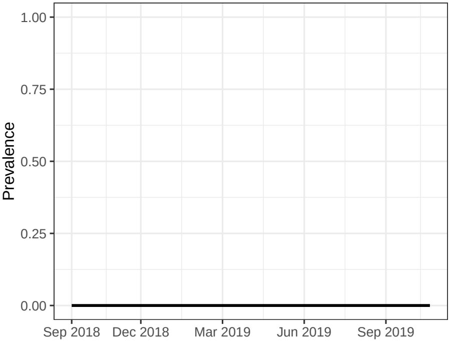

Supplement: Supplementary 2 — Figure S2: a 3-week centered moving average of weekly prevalence of CSF among wild boar within a 10-km radius of each farm. [file 1305664.f2.pdf]
