# Supplementary material for: Identifying Effective Biosecurity Measures for Preventing the Introduction of Classical Swine Fever in Pig Farms in Japan: Under the Condition of Absence/Presence of Observable Infected Wild Boar
Source: Transbound Emerg Dis. 2024 Jul 31;2024:1305664. doi: 10.1155/2024/1305664 (PMC12017048; doi:10.1155/2024/1305664)
Supplement: Supplementary 3 — Table S1: proportions of farms that meet the farm attributes and implementation rates of biosecurity measures. Table S2: results of univariable analyses using the prevalence and transmission kernel (TK) approaches during the non-wild boar (non-WB) phase. “NA” denotes a variable that was not estimated due to complete separation. Table S3: results of univariable analyses using the prevalence and transmission kernel (TK) approaches during the wild boar (WB) phase. “NA” denotes a variable that was not estimated due to complete separation. [file 1305664.f3.docx]

Table S1. Proportions of farms that meet the farm attributes and implementation rates of biosecurity measures.

| **Items** | **Number of farms that implemented biosecurity measures/number of farms that responded** | **Implementation rate (%)** |
| --- | --- | --- |
| **Farm information** |  |  |
| Non-adjacency to national or prefectural main roads | 36/51 | 70.6 |
| Absence of public roads on the farm | 18/53 | 34.0 |
| Not being located in a forest area | 32/53 | 60.4 |
| **Biosecurity practices** |  |  |
| Parking staff vehicles off farm site or disinfecting vehicles before entering the farm site | 31/53 | 58.5 |
| Properly adjusting the concentration of disinfectant for vehicles | 38/47 | 80.9 |
| Separating the entrances for employees’ vehicles/feed transport vehicles and compost/pig transport vehicles | 13/47 | 27.7 |
| Clarifying the border of the hygienic control area | 32/49 | 65.3 |
| Conducting showering-in | 14/49 | 28.6 |
| Changing into special clothing when entering the hygiene control area | 40/49 | 81.6 |
| Daily washing and disinfecting of special clothing for each pig house | 41/49 | 83.7 |
| Changing into special clothing for each pig house | 21/49 | 42.9 |
| Changing into special boots for each pig house | 39/49 | 79.6 |
| Establishing clear zones for where to place outer and inner boots | 41/49 | 83.7 |
| Disinfecting boots in a disinfection bath | 40/53 | 75.5 |
| Properly adjusting the concentration of disinfectants for disinfecting boots | 35/53 | 66.0 |
| Daily changing of disinfectant in the boot bath | 8/51 | 15.7 |
| Cleaning the dirt off boots before stepping into the disinfectant bath | 34/52 | 65.4 |
| Disinfecting hands or wearing hygienic gloves | 34/53 | 64.2 |
| Assignment of personnel for each pig development stage | 33/52 | 63.5 |
| No moving of pigs by having them walk on the ground | 32/53 | 60.4 |
| No use of mountain stream water for drinking water | 45/51 | 88.2 |
| No use of human food waste from meat processing facilities as feed | 47/51 | 92.2 |
| Absence of staff living on the farm premises | 44/53 | 83.0 |
| No employment of foreign trainees | 44/53 | 83.0 |
| No attending social gatherings with other swine producers | 39/52 | 75.0 |
| No entering of feed transport vehicle into hygienic control area | 12/52 | 23.1 |
| Contract with veterinarians who provide hygiene guidance | 48/52 | 92.3 |
| Disinfecting veterinarians’ vehicles | 45/50 | 90.0 |
| Changing into special boots by veterinarians | 53/53 | 100 |
| Cleaning veterinarians’ hands before providing veterinary services | 50/52 | 96.2 |
| Disinfecting construction tools transported onto the farm | 23/51 | 45.1 |
| Changing into special clothing by representatives of repair services | 39/52 | 75.0 |
| Changing into special boots by facility construction services | 47/52 | 90.4 |
| Disinfecting hands by facility construction services | 37/52 | 71.2 |
| Restricting entry of visitors not related to the farm | 48/53 | 90.6 |
| Restricting entry of hunters | 52/53 | 98.1 |
| No use of a common compost station | 44/53 | 83.0 |
| Preventing wildlife intrusion into areas used to store pig carcasses | 35/51 | 68.6 |
| No composting of pig carcasses on own farm | 28/48 | 58.3 |
| Contract with service for disposal of pig carcasses | 28/53 | 52.8 |
| Installation of perimeter fences around the farm | 35/52 | 66.0 |
| Height of perimeter fence surrounding the farm ≥ 1.5 m | 19/47 | 40.4 |
| Mesh size of perimeter fence surrounding the farm < 5 cm | 17/46 | 37.0 |
| Closing the entrance of the perimeter fence | 25/51 | 49.0 |
| Inspection of holes in the pig house walls | 11/51 | 21.6 |
| Preventing the intrusion of mice and other small wild animals into pig houses | 13/51 | 25.5 |
| Preventing the intrusion of cats, raccoons, and other medium-sized wild animals into the hygienic control area | 16/52 | 30.8 |
| Preventing the intrusion of feral cats, raccoons, and other medium-sized wild animals into the pig house | 32/52 | 61.5 |
| Installation of bird nets around the pig houses | 45/51 | 88.2 |
| Mesh size of bird net ≤ 2 cm | 21/47 | 44.7 |
| Preventing the intrusion of wild birds into the pig houses | 38/52 | 73.1 |

Table S2. Results of univariable analyses using the prevalence and transmission kernel (TK) approaches during the non-wild boar (non-WB) phase. “NA” denotes a variable that was not estimated due to complete separation.

| **Variables** | **Prevalence approach** | | **TK approach** | |
| --- | --- | --- | --- | --- |
|  | **Coefficient**  **(95% CI)** | ***p* value** | **Coefficient**  **(95% CI)** | ***p* value** |
| Non-adjacency to national or prefectural main road | 0.92 (-1.20, 3.05) | 0.359 | 0.98 (-2.25, 4.22) | 0.489 |
| Absence of public roads on the farm | 0.28 (-1.79, 3.29) | 0.809 | NA | NA |
| Absence of staff living on the farm premises | -0.48 (-2.54, 2.53) | 0.678 | -1.54 (-4.77, 1.70) | 0.279 |
| No employment of foreign trainees | -1.36 (-3.49, 0.78) | 0.178 | NA | NA |
| Not being located within or adjacent to a forest | -1.08 (-3.48, 1.13) | 0.317 | NA | NA |
| Parking staff vehicles off farm site or disinfecting vehicles before entering the farm site | -0.63 (-2.77, 1.51) | 0.537 | NA | NA |
| Properly adjusting the concentration of disinfectant for vehicles | -1.83 (-3.95, 0.30) | 0.068 | NA | NA |
| Separating the entrances for employees’ vehicles/feed transport vehicles and compost/pig transport vehicles | 0.13 (-2.88, 2.20) | 0.909 | 1.15 (-2.10, 4.40) | 0.422 |
| Clarifying the border of the hygienic control area | NA | NA | NA | NA |
| Conducting showering-in | -0.14 (-3.15, 1.92) | 0.903 | 1.03 (-2.20, 4.27) | 0.467 |
| Changing into special clothing when entering hygiene control area | NA | NA | NA | NA |
| Daily washing and disinfecting of special clothing for each pig house | -2.28 (-4.40, -0.16) | 0.023 | -2.28 (-5.52, 0.95) | 0.107 |
| Changing into special clothing for each pig house | -0.72 (-3.73, 1.33) | 0.531 | 0.33 (-2.90, 3.57) | 0.814 |
| Changing into special boots for each pig house | -0.02 (-2.15, 3.04) | 0.985 | NA | NA |
| Establishing clear zones for where to place outer and inner boots | -0.41(-2.52, 2.64) | 0.732 | NA | NA |
| Disinfecting boots in a disinfection bath | NA | NA | NA | NA |
| Properly adjusting the concentration of disinfectants for disinfecting boots | NA | NA | NA | NA |
| Daily changing disinfectant in the boot bath | 1.07 (-1.97, 3.25) | 0.371 | NA | NA |
| Cleaning the dirt off boots before stepping into the disinfectant bath | -0.69 (-2.83, 1.45) | 0.493 | NA | NA |
| Disinfecting hands or wearing hygienic gloves | 0.62 (-1.44, 3.63) | 0.593 | NA | NA |
| Assignment of personnel for each pig development stage | 0.35 (-1.71, 3.36 | 0.761 | NA | NA |
| Not moving pigs by having them walk on the ground | 1.00 (-1.08, 4.03) | 0.391 | NA | NA |
| No use of mountain stream water for drinking water | NA | NA | NA | NA |
| No use of human food waste from meat processing facilities as feed | NA | NA | NA | NA |
| No attending social gatherings with other swine producers | -0.41 (-2.46, 2.60) | 0.724 | NA | NA |
| No entering of feed transport vehicle into hygienic control area | NA | NA | NA | NA |
| Contract with veterinarians who provide hygiene guidance | -1.60 (-3.74, 1.43) | 0.175 | NA | NA |
| Cleaning veterinarians’ hands before providing veterinary services | -0.98 (-3.05, 2.04) | 0.399 | NA | NA |
| Disinfecting construction tools transported onto the farm | NA | NA | NA | NA |
| Changing into special clothing by representatives of repair services | -0.09 (-2.15, 2.92) | 0.940 | NA | NA |
| Changing into special boots by facility construction services | -1.39 (-3.45, 1.62) | 0.232 | NA | NA |
| Disinfecting hands by facility construction services | 0.32 (-1.75, 3.34) | 0.783 | NA | NA |
| Restricting entry of visitors not related to the farm | NA | NA | NA | NA |
| No use of a common compost station | NA | NA | NA | NA |
| Preventing wildlife intrusion into areas used to store pig carcasses | 0.87 (-1.24, 3.92) | 0.465 | NA | NA |
| No composting of pig carcasses on own farm | 0.98 (-1.14, 4.04) | 0.414 | NA | NA |
| Contract with service for disposal of pig carcasses | -0.31 (-2.44, 1.83) | 0.761 | NA | NA |
| Installation of perimeter fences around the farm | -1.62 (-4.00, 0.59) | 0.131 | NA | NA |
| Height of perimeter fence surrounding the farm ≥ 1.5 m | 0.43 (-1.70, 2.56) | 0.669 | 0.32 (-2.93, 3.57) | 0.822 |
| Mesh size of perimeter fence surrounding the farm < 5 cm | 0.37 (-1.79, 2.52) | 0.716 | NA | NA |
| Closing the entrance of the fence | NA | NA | NA | NA |
| Inspection of holes in the pig house walls | 1.59 (-0.55, 3.72) | 0.115 | 1.45 (-1.84, 4.72) | 0.318 |
| Preventing the intrusion of mice and other small wild animals into pig houses | 0.12 (-2.89, 2.18) | 0.916 | 1.18 (-2.05, 4.41) | 0.403 |
| Preventing the intrusion of cats, raccoons, and other medium-sized wild animals in the hygienic control area | 1.89 (-0.16, 4.90) | 0.101 | NA | NA |
| Preventing the intrusion of feral cats, raccoons, and other medium-sized wild animals into the pig house | NA | NA | NA | NA |
| Installation of bird nets around the pig houses | NA | NA | NA | NA |
| Mesh size of bird net ≤ 2 cm | -0.66 (-3.67, 1.44) | 0.574 | 0.73 (-2.58, 4.10) | 0.624 |
| Preventing the intrusion of wild birds into the pig houses | 0.35 (-1.71, 3.35) | 0.765 | NA | NA |

Table S3. Results of univariable analyses using the prevalence and transmission kernel (TK) approaches during the wild boar (WB) phase. “NA” denotes a variable that was not estimated due to complete separation.

| **Variables** | **Prevalence approach** | | **TK approach** | |
| --- | --- | --- | --- | --- |
|  | **Coefficient**  **(95% CI)** | ***p* value** | **Coefficient**  **(95% CI)** | ***p* value** |
| Non-adjacency to national or prefectural main road | -0.04 (-1.06, 0.93) | 0.941 | -0.07 (-1.09, 0.89) | 0.889 |
| Absence of public roads on the farm | -0.73 (-1.77, 0.40) | 0.181 | -0.69 (-1.68, 0.35) | 0.175 |
| Absence of staff living on the farm premises | -0.47 (-1.66, 1.04) | 0.477 | -0.50 (-1.66, 0.99) | 0.443 |
| No employment of foreign trainees | -1.26 (-2.90, 1.64) | 0.227 | -1.22 (-2.85, 1.69) | 0.242 |
| Not being located within or adjacent to a forest | 0.00 (-1.07, 0.98) | 0.999 | -0.06 (-1.15, 0.93) | 0.905 |
| Parking staff vehicles off farm site or disinfecting vehicles before entering the farm site | 0.75 (-0.23, 1.75) | 0.130 | 0.55 (-0.42, 1.54) | 0.259 |
| Properly adjusting the concentration of disinfectant for vehicles | -0.45 (-1.51, 0.83) | 0.440 | -0.88 (-1.81, 0.17) | 0.076 |
| Separating the entrances for employees’ vehicles/feed transport vehicles and compost/pig transport vehicles | -0.38 (-1.45, 0.60) | 0.463 | -0.51 (-1.56, 0.42) | 0.300 |
| Clarifying the border of the hygienic control area | 0.47 (-0.59, 1.76) | 0.417 | 0.75 (-0.37, 2.08) | 0.223 |
| Conducting showering-in | 0.44 (-0.65, 1.43) | 0.401 | 0.32 (-0.75, 1.29) | 0.524 |
| Changing into special clothing when entering hygiene control area | -0.19 (-1.23, 1.08) | 0.737 | 0.06 (-0.99, 1.33) | 0.924 |
| Daily washing and disinfecting of special clothing for each pig house | 0.45 (-0.72, 1.95) | 0.494 | 0.25 (-0.86, 1.60) | 0.684 |
| Changing into special clothing for each pig house | -0.18 (-1.19, 0.84) | 0.719 | -0.31 (-1.26, 0.62) | 0.504 |
| Changing into special boots for each pig house | 0.34 (-0.94, 2.20) | 0.653 | 0.22 (-0.92, 1.70) | 0.734 |
| Establishing clear zones for where to place outer and inner boots | -0.34 (-1.71, 1.55) | 0.665 | -0.47 (-1.59, 1.00) | 0.460 |
| Disinfecting boots in a disinfection bath | 1.31 (0.18, 2.79) | 0.040 | 2.13(0.73, 3.90) | 0.007 |
| Properly adjusting the concentration of disinfectants for disinfecting boots | 1.24 (0.19, 2.51) | 0.032 | 2.17 (0.81, 3.89) | 0.005 |
| Daily changing disinfectant in the boot bath | 0.48 (-0.81, 1.56) | 0.419 | 0.76 (-0.43, 1.83) | 0.176 |
| Cleaning the dirt off boots before stepping into the disinfectant bath | 1.03 (-0.02, 2.31) | 0.074 | 0.90 (-0.16, 2.13) | 0.114 |
| Disinfecting hands or wearing hygienic gloves | 0.50 (-0.55, 1.78) | 0.386 | 0.54 (-0.45, 1.69) | 0.312 |
| Assignment of personnel for each pig development stage | 0.47 (-0.53, 1.62) | 0.382 | 0.54 (-0.60, 1.88) | 0.384 |
| Not moving pigs by having them walk on the ground | 0.58 (-0.47, 1.86) | 0.316 | 0.57 (-0.44, 1.75) | 0.300 |
| No use of mountain stream water for drinking water | -0.39 (-1.44, 0.88) | 0.499 | -0.50 (-1.49, 0.65) | 0.346 |
| No use of human food waste from meat processing facilities as feed | 1.38 (-0.26, 4.30) | 0.190 | 1.86 (0.10, 4.82) | 0.090 |
| No attending social gatherings with other swine producers | 0.96 (-0.09, 2.23) | 0.096 | 0.92 (-0.07, 2.07) | 0.088 |
| No entering of feed transport vehicle into hygienic control area | -0.66 (-2.14, 0.49) | 0.311 | -0.89 (-2.37, 0.26) | 0.168 |
| Contract with veterinarians who provide hygiene guidance | NA | NA | 0.95 (-0.68, 3.86) | 0.362 |
| Cleaning veterinarians’ hands before providing veterinary services | -0.09 (-1.79, 2.84) | 0.936 | -0.39 (-1.68, 1.47) | 0.605 |
| Disinfecting construction tools transported onto the farm | -0.16 (-1.15, 0.87) | 0.754 | -0.30 (-1.26, 0.65) | 0.528 |
| Changing into special clothing by representatives of repair services | 0.63 (-0.54, 2.13) | 0.341 | 0.64 (-0.49, 1.99) | 0.304 |
| Changing into special boots by facility construction services | 0.25 (-1.43, 3.17) | 0.812 | -0.24 (-1.52, 1.62) | 0.755 |
| Disinfecting hands by facility construction services | 0.58 (-0.56, 2.06) | 0.369 | 0.63 (-0.51, 1.98) | 0.316 |
| Restricting entry of visitors not related to the farm | -0.26 (-1.57, 1.61) | 0.732 | -0.09 (-1.36, 1.76) | 0.905 |
| No use of a common compost station | -1.11 (-2.75, 1.79) | 0.287 | -1.00 (-2.64, 1.90) | 0.336 |
| Preventing wildlife intrusion into areas used to store pig carcasses | -0.95 (-2.04, 0.35) | 0.106 | -1.00 (-2.03, 0.18) | 0.069 |
| No composting of pig carcasses on own farm | -0.65 (-1.61, 0.34) | 0.186 | -0.74 (-1.71, 0.23) | 0.127 |
| Contract with service for disposal of pig carcasses | -0.20 (-1.35, 0.79) | 0.705 | -0.42 (-1.57, 0.56) | 0.429 |
| Installation of perimeter fences around the farm | -0.12 (-1.61, 1.86) | 0.884 | 0.12 (-1.25, 2.04) | 0.882 |
|  |  |  |  |  |
| Height of perimeter fence surrounding the farm ≥ 1.5 m | -0.83 (-2.13, 0.25) | 0.156 | -0.71 (-1.86, 0.26) | 0.176 |
| Mesh size of perimeter fence surrounding the farm < 5 cm | -0.69 (-2.16, 0.43) | 0.280 | -0.94 (-2.40, 0.17) | 0.137 |
| Closing the entrance of the fence | 0.16 (-0.88, 1.36) | 0.769 | 0.44 (-0.63, 1.66) | 0.446 |
| Inspection of holes in the pig house walls | -0.43 (-1.74, 0.68) | 0.470 | -0.32 (-1.47, 0.67) | 0.543 |
| Preventing the intrusion of mice and other small wild animals into pig houses | -0.58 (-1.87, 0.51) | 0.326 | -0.63 (-1.90, 0.41) | 0.269 |
| Preventing the intrusion of cats, raccoons, and other medium-sized wild animals in the hygienic control area | -0.06 (-1.37, 1.06) | 0.924 | 0.20 (-1.01, 1.30) | 0.730 |
| Preventing the intrusion of feral cats, raccoons, and other medium-sized wild animals into the pig house | -0.12 (-1.13, 1.03) | 0.818 | 0.05 (-0.93, 1.19) | 0.920 |
| Installation of bird nets around the pig houses | 0.25 (-1.43, 3.17) | 0.812 | 0.42 (-1.20, 3.33) | 0.687 |
| Mesh size of bird net ≤ 2 cm | -0.69 (-1.71, 0.37) | 0.187 | -0.83 (-1.81, 0.14) | 0.088 |
| Preventing the intrusion of wild birds into the pig houses | -0.66 (-2.09, 1.27) | 0.419 | -0.75 (-1.92, 0.76) | 0.258 |
